# Bionic adaptive fault-tolerant control of non-Gaussian stochastic attitude hypersonic vehicle

**DOI:** 10.1038/s41598-022-24138-0

**Published:** 2022-11-15

**Authors:** Kai-Yu Hu, Kun Zhu, Wenjing Sun

**Affiliations:** Aviation Technology Research Institute, China Aerospace Science and Industry Corporation, Beijing, 100143 China

**Keywords:** Engineering, Mathematics and computing

## Abstract

This study investigates an adaptive fault-tolerant control (FTC) for hypersonic flight vehicles (HFVs) with incipient faults and non-Gaussian stochastic output attitudes. In the nonlinear HFV dynamics, a hybrid fuzzy approximation method achieves the linearization, then the stochastic outputs are transformed into probability density functions (PDFs) via rational square root B-spline. The disturbance and faults are estimated simultaneously by an adaptive augmented observer. Then actuator faults are compensated by an bionic adaptive fault-tolerant controller to ensure that the output PDFs accurately track the expected PDFs, thereby matching actual attitude angles with the desired ones, the bionic prey adaptive law can make FTC accurately repair the incipient fault deviations. Lyapunov theory proves the robust stability of the scheme, and simulation illustrates the effectiveness.

## Introduction

In recent years, some new advances in modelling, fault estimation and fault-tolerant control (FTC) for hypersonic flight vehicle (HFV) or other kinds of spacecrafts have been achieved^1–5^. Where the study of uncertainties can improve the algorithm reliability, which mainly focus on the uncertainty of parameters, actuator faults and environment. In^6,7^, the disturbance observer (DOB) was investigated to deal with the HFV system with disturbance or uncertain wind effects. Various subsystems of HFV have a variety of fault types^8–10^. In^11^, an estimation algorithm was proposed to estimate the time-varying bias and gain faults. In^12^, a fault tolerant strategy was designed for the re-entry HFV with mixed aerodynamic surfaces under uncertainties and faults. In^13,14^, a novel adaptive FTC was proposed for HFV with input constraints and uncertain parameters. This paper simultaneously studies the FTC method for the approximated nonlinear HFV with attitude output uncertainties and actuator faults.

Laser weapons can counter HFV but require time to accumulate energy to burn obstacles^15,16^. Hence, one anti-laser method is attitude active randomization. Output uncertainty should also be investigated because of the harsh environment. Given these uncertain outputs, FTC for non-Gaussian stochastic systems can improve the HFV reliability, and linear B-spline interpolation is a method to obtain stochastic distributions^17^. Other complex interpolations meet the high reliability are also useful. For example in^18^, the rational square root algorithm was presented for the non-Gaussian output probability density function (PDF).

New progress is achieved in the reconstruction and tracking control of systems with singularity, time-delay functions and non-Gaussian stochastic outputs. Complex fuzzy approximation theory is an effective tool for solving complex nonlinear problems^19,20^. In^21^, T–S fuzzy theory was used for non-Gaussian stochastic systems, whilst a sliding-mode algorithm compensated for the fault impacts. In^22^, the measurable premise variables and estimations of the immeasurable premise variables were used for fuzzy controllers. In^23,24^, the state feedback robust controller and output feedback dynamic control strategy were designed for complex singular systems. In^25^, algorithms were proposed for the singular time-delay systems with non-Gaussian stochastic output and PDF approximation error. The time delay problem was investigated in^26,27^, in which the adaptive-robust delay controllers were used to solve the problem of the Euler–Lagrange systems. An adaptive observer and fault-tolerant PI controller were designed because of the singular and delay of non-Gaussian systems^28^. In^29^, the enhanced robust observer and neurocontrollers were presented for the systems with output uncertainties to repair faults and shield disturbance. The main considerations in this paper include fuzzy linearization, singularity, exogenous interference and non-Gaussian stochastic FTC.

We use PDF as the algorithm variable to enable the controller to receive accurate internal information of the attitude angles, then the output PDF shape can perfectly match the expected value when the fault occurs, finally the FTC of HFV with non-Gaussian stochastic attitudes is realized. The contributions and innovations of this study are as follows:

1. Proposing a non-Gaussian stochastic HFV system to solve the problem of stochastic attitude angle modelling and real-time statistics of the distribution information. Displaying the fault information using PDF deformations is more accurate than the conventional attitude angle signals.
2. Proposing a multi-fuzzy approximation scheme to linearize the HFV and disturbance interconnect systems, then designing an adaptive DOB and proving the robust stability.
3. Designing an improved adaptive fuzzy fault estimation and FTC scheme by mimicking animal predation behavior, which are better than the traditional method when diagnosing and compensating for incipient faults.

Section 2 introduces the HFV model with non-Gaussian stochastic attitude angles. Section 3.1 presents an augmented estimation observer to estimate the incipient deviation fault and disturbance. Section 3.2 proposes a improved adaptive compensation controller. Section 3.3 designs the bionic prey adaptive law. Lastly, Sect. 4 verifies the methods.

## System and fault models

**Figure 1:**
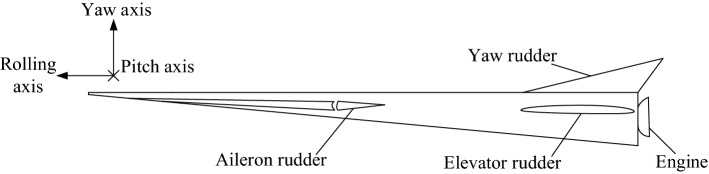
Simplified structure of HFV.

The rudders of HFV are shown in Fig. 1. The stochastic attitudes are generated by the deterministic rudder commands combining with uncertain environment or stochastic jet rudders. Fixed rudders can achieve stochastic attitude tracking control.

With reference to^14^, the following reentry HFV model with non-Gaussian stochastic attitude angles is designed:

1 $$ \begin{aligned} & E\dot{x}(t) = A(x(t))x(t) + A_{d} (x(t - \tau (t)))x(t - \tau (t)) \\ & \quad \quad \quad + B(x(t))u(t) + NF_{com} (t) + B_{d} d(t) \\ & x(t) = \phi (t),t \in [ - \iota ,0] \\ & V(t) = D(\Xi x(t)) \\ & \gamma (\rho ,u(t)) = \Phi^{2} (C(\rho )V(t))^{2} \\ \end{aligned} $$

where $$x(t) = [\begin{array}{*{20}c} {\overline{p}(t)} & {\overline{q}(t)} & {\overline{r}(t)} \\ \end{array} ]^{T}$$ and $\overline{p}(t)$, $\overline{q}(t)$ and $\overline{r}(t)$ are the pitch, roll and yaw rates, respectively; $$\rho = [\begin{array}{*{20}c} {\rho_{1} } & {\rho_{2} } & {\rho_{3} } \\ \end{array} ]^{T}$$ and *ρ*_1_, *ρ*_2_ and *ρ*_3_ are the bank, sideslip, attack angles, respectively; $$u(t) = [\begin{array}{*{20}c} {\delta_{e} } & {\delta_{a} } & {\delta_{r} } \\ \end{array} ]^{T}$$ is the control surface deflection and *δ*_*e*_, *δ*_*a*_ and *δ*_*r*_ are the elevator, aileron and yaw rudder deflections, respectively; *V*(*t*) ∈ *R*^3^ is the weight vector and *τ*(*t*) is the time-varying delay function that satisfies $0 \le \tau (t) \le \iota$; $\phi (t)$ is an initial rudder rate function and *F*_*com*_(*t*) ∈ *R*^3^ is the rudder fault vector and set *F*_*com*_(*t*) as follows:

2 $$ F_{com} (t) \subseteq \{ F_{1} (t), F(t)\} $$

*F*1(*t*) is the incipient rudder deviation satisfying (3) with reference to^30^. *F*(*t*) is the standard deviation fault, which does not meet (3).

3 $$ \left\| {F_{1} (t)/u(t)} \right\| \le 10\% $$

The incipient deviation is characterised with three features^30^: (1) from the qualitative aspect, the deteriorated degree is insufficient to trigger any pre-set fault alarms; (2) from the quantitative aspect, the deviation percent ranks from 1 to 10%, these slight abnormalities are easily affected by the systems; (3) it will develop into a catastrophic fault or the fault like *F*(*t*).

$A(x(t))$ and $B(x(t)) \in R^{3 \times 3}$ are the nonlinear matrix functions that satisfy the following equations:

4 $$ A(x(t)) = J^{ - 1} \Theta (x(t))J $$

5 $$ B(x(t)) = J^{ - 1} G $$

where *J* ∈ *R*^3×3^ is the inertia matrix, *G* is the control allocation matrix distributes the control torque to the control surfaces:

$$ \Theta (x(t)) = \left[ {\begin{array}{*{20}c} 0 & {\overline{r}(t)} & { - \overline{q}(t)} \\ { - \overline{r}(t)} & 0 & {\overline{p}(t)} \\ {\overline{q}(t)} & { - \overline{p}(t)} & 0 \\ \end{array} } \right],\quad G = \left[ {\begin{array}{*{20}c} {g_{{p,\delta_{e} }} } & {g_{{p,\delta_{a} }} } & {g_{{p,\delta_{r} }} } \\ {g_{{q,\delta_{e} }} } & {g_{{q,\delta_{a} }} } & {g_{{q,\delta_{r} }} } \\ {g_{{r,\delta_{e} }} } & {g_{{r,\delta_{a} }} } & {g_{{r,\delta_{r} }} } \\ \end{array} } \right]. $$

*E*, *B*_*d*_, *N* ∈ *R*^3×3^ are the parameter matrices, where *rank*(*E*) = *q* < 3. $D( \cdot ) \in R^{3 \times 3}$ is a transformation matrix function contains an equivalent integral and a linear transformation functions. $$\Xi x(t) = [\begin{array}{*{20}c} {\dot{\rho }_{1} } & {\dot{\rho }_{2} } & {\dot{\rho }_{3} } \\ \end{array} ]^{T}$$ represents the angular rates and Ξ satisfies (6).

6 $$ \Xi = \left[ {\begin{array}{*{20}c} {\cos \rho_{3} } & 0 & {\sin \rho_{3} } \\ {\sin \rho_{3} } & 0 & { - \cos \rho_{3} } \\ 0 & 1 & 0 \\ \end{array} } \right] $$

Output (7) describes the non-Gaussian stochastic attitude angles, whilst its PDFs are approximated by the rational square root B-spline functions. The re-entry HFV model has three outputs. Thus,

7 $$ \begin{gathered} \gamma (\rho ,u(t)) = \left[ {\begin{array}{*{20}c} {\gamma_{1} (\rho_{1} ,u(t))} & {\gamma_{2} (\rho_{2} ,u(t))} & {\gamma_{3} (\rho_{3} ,u(t))} \\ \end{array} } \right]^{T} \hfill \\ {\kern 1pt} {\kern 1pt} {\kern 1pt} {\kern 1pt} {\kern 1pt} {\kern 1pt} {\kern 1pt} {\kern 1pt} {\kern 1pt} {\kern 1pt} {\kern 1pt} {\kern 1pt} {\kern 1pt} {\kern 1pt} {\kern 1pt} {\kern 1pt} {\kern 1pt} {\kern 1pt} {\kern 1pt} {\kern 1pt} {\kern 1pt} {\kern 1pt} {\kern 1pt} {\kern 1pt} {\kern 1pt} {\kern 1pt} {\kern 1pt} {\kern 1pt} {\kern 1pt} {\kern 1pt} {\kern 1pt} {\kern 1pt} {\kern 1pt} {\kern 1pt} {\kern 1pt} {\kern 1pt} {\kern 1pt} {\kern 1pt} {\kern 1pt} {\kern 1pt} {\kern 1pt} {\kern 1pt} {\kern 1pt} {\kern 1pt} {\kern 1pt} {\kern 1pt} {\kern 1pt} {\kern 1pt} {\kern 1pt} = \left[ {\begin{array}{*{20}c} {\Phi^{2} (C_{\rho 1} V(t))^{2} } & {\Phi^{2} (C_{\rho 2} V(t))^{2} } & {\Phi^{2} (C_{\rho 3} V(t))^{2} } \\ \end{array} } \right]^{T} \hfill \\ \end{gathered} $$

where

$$ \begin{aligned} & C(\rho ) = \left[ {\begin{array}{*{20}c} {C_{\rho 1} } \\ {C_{\rho 2} } \\ {C_{\rho 3} } \\ \end{array} } \right] = \left[ {\begin{array}{*{20}c} {\varphi_{11} (\rho_{1} )} & \ldots & {\varphi_{13} (\rho_{1} )} \\ {\varphi_{21} (\rho_{2} )} & \ldots & {\varphi_{23} (\rho_{2} )} \\ {\varphi_{31} (\rho_{3} )} & \ldots & {\varphi_{33} (\rho_{3} )} \\ \end{array} } \right], \\ & V = [w_{1} ,{\kern 1pt} \ldots ,w_{3} ]^{T} , \\ & \Phi = 1/\sqrt {V^{T} (t)\Sigma_{1} V(t)} , \\ & \Sigma_{1} = \int_{a}^{b} {C^{T} (\rho )C(\rho )d\rho } . \\ \end{aligned} $$

And $\varphi_{{i\tilde{i}}} (\rho )(i = 1,2,3,\tilde{i} = 1,2,3)$ are the predetermined basis functions; *w*_*i*_ are the tracking weights; *γ*_1_, *γ*_2_ and *γ*_3_ are the PDFs of the bank, sideslip and attack angles, respectively. Compared with the traditional models, (1) describes the microscopic stochastic details of the output attitude angles. The PDF deformation can display additional information that the classic outputs cannot show using mean and variance, thereby improving control accuracy.

### *Remark 1*

The mean of the non-Gaussian attitude angles reveals the overall performance and is an important reference for the HFV full-process tracking control and task execution. Our model can calculate and control the mean in real time using (8). Arithmetic * is the matrix Hadamard product.

8 $$ \mu_{mean} = \int_{a}^{b} {\rho * \gamma (\rho ,u(t))} d\rho $$

The nonlinear external disturbance is expressed as follows:

9 $$ \left\{ \begin{gathered} \dot{\omega }(t) = \Omega (t)\omega (t) \hfill \\ d(t) = T(t)\omega (t) \hfill \\ \end{gathered} \right. $$

where $\omega (t),{\kern 1pt} {\kern 1pt} {\kern 1pt} d(t) \in R^{3 \times 1}$, $T(t),{\kern 1pt} {\kern 1pt} {\kern 1pt} \Omega (t) \in R^{3 \times 3}$, $\Omega (t)$ is the functions describe the distance between disturbance source and HFV.

Systems (1) is linearised using double fuzzy approximation. The *i*th rule: $IF\;\varpi_{1} (t)\;{\kern 1pt} is\;{\kern 1pt} \beta_{i1} ,and \ldots ,and\;\varpi_{\sigma } (t)\;is\;\beta_{i\sigma } ,\;THEN$:

10 $$ \begin{aligned} & E_{i} \dot{x}(t) = A_{i} x(t) + A_{id} x(t - \tau (t)) + B_{i} u(t) + N_{i} F_{com} (t) + B_{id} d(t) \\ & V(t) = D_{i} \Xi_{i} x(t) \\ & x(t) = \phi (t),\quad t \in [ - \iota ,0] \\ & \gamma (\rho ,u(t)) = \Phi^{2} (C(\rho )V(t))^{2} \\ \end{aligned} $$

The *w*th rule: $IF{\kern 1pt} {\kern 1pt} {\kern 1pt} \Delta_{1} (t){\kern 1pt} {\kern 1pt} {\kern 1pt} is{\kern 1pt} {\kern 1pt} {\kern 1pt} \beta_{w1} ,and...,and{\kern 1pt} {\kern 1pt} {\kern 1pt} \Delta_{\upsilon } (t){\kern 1pt} {\kern 1pt} {\kern 1pt} is{\kern 1pt} {\kern 1pt} {\kern 1pt} \beta_{w\upsilon } ,{\kern 1pt} {\kern 1pt} {\kern 1pt} THEN:$

11 $$ \left\{ \begin{gathered} \dot{\omega }(t) = \Omega_{w} \omega (t) \hfill \\ d(t) = T_{w} \omega (t) \hfill \\ \end{gathered} \right. $$

where in (10), $\varpi_{j} (j = 1,...,\sigma )$ are the prerequisite variables and $\beta_{ij} (i = 1,...,\overline{n})$ are the fuzzy sets; in (11), $\Delta_{\vartheta } (\vartheta = 1,...,\upsilon )$ are the prerequisite variables and $\beta_{w\vartheta } (w = 1,...,\overline{m})$ are the fuzzy sets.

12 $$ \left\{ \begin{gathered} h_{i} (\varpi (t)) = \prod\limits_{j = 1}^{\sigma } {\beta_{ij} (\varpi (t))} /\sum\limits_{i = 1}^{{\overline{n}}} {\prod\limits_{j = 1}^{\sigma } {\beta_{ij} (\varpi (t))} } \ge 0 \hfill \\ h_{w} (\Delta (t)) = \prod\limits_{\vartheta = 1}^{\upsilon } {\beta_{w\vartheta } (\Delta (t))} /\sum\limits_{w = 1}^{{\overline{m}}} {\prod\limits_{\vartheta = 1}^{\upsilon } {\beta_{w\vartheta } (\Delta (t))} } \ge 0 \hfill \\ \sum\limits_{i = 1}^{{\overline{n}}} {h_{i} (\varpi (t))} = \sum\limits_{w = 1}^{{\overline{m}}} {h_{w} (\Delta (t))} = 1 \hfill \\ \end{gathered} \right. $$

**Figure 2:**
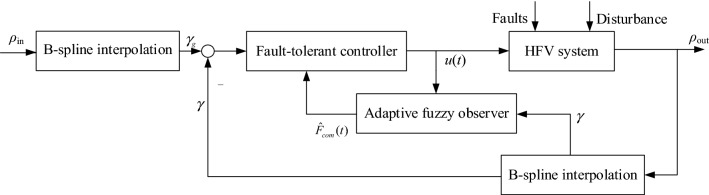
Block diagram of fault-tolerant control.

Figure 2 shows the overall block diagram of the HFV system fault-tolerant scheme.

### *Assumption 1*

$\left\| {F_{1} (t)} \right\|,\left\| {F(t)} \right\| \le M_{f}$, $\left\| {d(t)} \right\| \le M_{d}$. *M*_*f*_ and *M*_*d*_ are two positive scalars.

### *Assumption 2*

There exist two reversible matrices *L*_1_ and *L*_2_ such that: *L*_*i*1_*A*_*id*_*L*_*i*2_ = [*A*_*id*1_ 0; 0 *A*_*id*2_], *L*_*i*1_*E*_*i*_*L*_*i*2_ = [*I*_*q*_ 0; 0 0], *L*_*i*1_*A*_*i*_*L*_*i*2_ = [*A*_*i*1_ 0; 0 *I*_3*−q*_].

### *Remark 2*

The disturbance source cannot have infinite power, so the amplitude of the electromagnetic wave transmitted by it is bounded. Therefore, setting bounded external disturbance can better reflect the real war scene. The rudders of HFV have a software and hardware dual limiting scheme. Even if there is a very small probability that breaks the two limits, the aerodynamic effect of the deflection angle beyond 180 deg is the same as the effect of the remaining angle after subtracting 180 deg. Therefore, it is reasonable to set the rudder fault to be bounded. In engineering, the decoupled HFV system reflects a flight environment with directional disturbance, it slows down the response speed of some control channels, while the control channels facing away from the disturbance source or away from the interference direction still maintain fast response. Hence the undisturbed channel is decoupled from the other channels. For example, the roll rudders is another set of independent execution system, if it is undisturbed, it is decoupled obviously. Therefore, it is reasonable to set up a singular HFV system that can be uncoupled.

Given the singularity and Assumption 2, systems (10) and (11) can be transformed as follows:

13 $$ \begin{aligned} \dot{x}_{1} (t) & = \sum\limits_{i = 1}^{{\overline{n}}} {\sum\limits_{w = 1}^{{\overline{m}}} {h_{i} (\varpi (t))h_{w} (\Delta (t))[A_{i1} x_{1} (t) + A_{id1} x_{1} (t - \tau (t))} } \\ & \quad + B_{i1} u(t) + N_{i1} F_{com} (t) + B_{id1} T_{w} \omega (t)] \\ x_{2} (t) & = \sum\limits_{i = 1}^{{\overline{n}}} {\sum\limits_{w = 1}^{{\overline{m}}} {h_{i} (\varpi (t))h_{w} (\Delta (t))[ - A_{id2} x_{2} (t - \tau (t)) - B_{i2} u(t)} } \\ & \quad - N_{i2} F_{com} (t) - B_{id2} T_{w} \omega (t)] \\ V(t) & = \sum\limits_{i = 1}^{{\overline{n}}} {\sum\limits_{w = 1}^{{\overline{m}}} {h_{i} (\varpi (t))h_{w} (\Delta (t))} } (D_{i1} \Xi_{i1} x_{1} (t) + D_{i2} \Xi_{i2} x_{2} (t)) \\ \dot{\omega }(t) & = \sum\limits_{i = 1}^{{\overline{n}}} {\sum\limits_{w = 1}^{{\overline{m}}} {h_{i} (\varpi (t))h_{w} (\Delta (t))} } \Omega_{w} \omega (t) \\ \end{aligned} $$

where *B*_*i*1_, *B*_*id*1_, *N*_*i*1_ ∈ *R*^*q*×3^, *B*_*i*2_, *B*_*id*2_, *N*_*i*2_ ∈ *R*^(3*-q*)×3^, *D*_*i*1_Ξ_*i*1_ ∈ *R*^3×*q*^, *D*_*i*2_Ξ_*i*2_ ∈ *R*^3×(3-*q*)^, which satisfies: *L*_*i*2_*B*_*i*_ = [*B*_*i*1_; *B*_*i*2_], *L*_*i*2_*B*_*id*_ = [*B*_*id*1_; *B*_*id*2_], *D*_*i*_Ξ_*i*_*L*_*i*1_ = [*D*_*i*1_Ξ_*i*1_ *D*_*i*2_Ξ_*i*2_], *L*_*i*2_*N*_*i*_ = [*N*_*i*1_; *N*_*i*2_].

This study is dedicated to providing the fault-tolerant control scheme for the faults with different deviation amplitudes in the HFV with stochastic attitude angles. The following sections are the scheme design process.

## Fault compensation control scheme

### Fault estimation

Disturbance and singularity lead to system uncertainty and inconsistent response. Disturbance can contaminate useful signals to severely distort system information, and the observer cannot accurately estimate faults, which can lead to instability. Singularity is an inherent property. Neglecting singularity can lead to poor tracking performance of the control scheme and difficult to be widely applied at the engineering site.

To solve these problems, two state error functions of fast-slow subsystems and one disturbance error function are constructed. By substituting these three functions into the performance indicators, new methods ensure that the system is H∞ stable.

#### *Definition 1*

To simplify the expression, we set the double fuzzy approximation superposition function shown in system (13) to satisfy:

14 $$ \Lambda_{(i,w)} (\varpi ,\Delta ) = \sum\limits_{i = 1}^{{\overline{n}}} {\sum\limits_{w = 1}^{{\overline{m}}} {h_{i} (\varpi (t))h_{w} (\Delta (t))} } $$

If there is only one type of linear weight mode in a double fuzzy approximation process, (14) will degenerate into a single fuzzy approximation superposition function, that is, satisfies:

15 $$ \Lambda_{(i,w)} (\varpi ,\Delta ) = \sum\limits_{i = 1}^{{\overline{n}}} {h_{i} (\varpi (t))} {\kern 1pt} {\kern 1pt} {\kern 1pt} {\kern 1pt} {\kern 1pt} or{\kern 1pt} {\kern 1pt} {\kern 1pt} {\kern 1pt} {\kern 1pt} \sum\limits_{w = 1}^{{\overline{m}}} {h_{w} (\Delta (t))} $$

This definition enables the simplification and unification of symbols in the derivation process.

The following system is obtained by combining systems (1) and (9) and letting $$\varsigma (t) = [\begin{array}{*{20}c} {x_{1}^{T} (t)} & {\omega^{T} (t)} \\ \end{array} ]^{T}$$:

16 $$ \begin{aligned} \dot{\varsigma }(t) & = \Lambda_{(i,w)} (\varpi ,\Delta )[A_{iw0} \varsigma (t) + A_{id0} \varsigma (t - \tau (t)) + B_{i0} u(t) + N_{i0} F_{com} (t)] \\ x_{2} & = \Lambda_{(i,w)} (\varpi ,\Delta )[ - A_{id2} x_{2} (t - \tau (t)) - B_{i2} u(t) - N_{i2} F_{com} (t) - B_{id2} T_{w} \omega (t)] \\ V(t) & = \Lambda_{(i,w)} (\varpi ,\Delta )(D_{i1} \Xi_{i1} x_{1} (t) + D_{i2} \Xi_{i2} x_{2} (t)) \\ & = \Lambda_{(i,w)} (\varpi ,\Delta )(D_{i0} \Xi_{i0} \varsigma (t) + D_{i2} \Xi_{i2} x_{2} (t)) \\ \end{aligned} $$

where *A*_*iw*0_ = [*A*_*i*1_ *B*_*id*1_*T*_*w*_; 0 Ω_*w*_], *A*_*id*0_ = [*A*_*id*1_ 0; 0 0], *B*_*i*0_ = [*B*_*i*1_; 0], *N*_*i*0_ = [*N*_*i*1_; 0] and *D*_*i*0_Ξ_*i*0_ = [*D*_*i*1_Ξ_*i*1_ 0]. The full-order DOB is designed as follows:

17 $$ \begin{aligned} \dot{\hat{\varsigma }}(t) & = \Lambda_{(i,w)} (\varpi ,\Delta )[A_{iw0} \hat{\varsigma }(t) + A_{id0} \hat{\varsigma }(t - \tau (t)) + B_{i0} u(t) + K_{i1} \varepsilon (t)] \\ \hat{x}_{2} & = \Lambda_{(i,w)} (\varpi ,\Delta )[ - A_{id2} \hat{x}_{2} (t - \tau (t)) - B_{i2} u(t) - N_{i2} F_{com} (t) - B_{id2} T_{w} \hat{\omega }(t)] \\ \hat{V}(t) & = \Lambda_{(i,w)} (\varpi ,\Delta )(D_{i0} \Xi_{i0} \hat{\varsigma }(t) + D_{i2} \Xi_{i2} \hat{x}_{2} (t)) \\ \varepsilon (t) & = \int_{a}^{b} {(\sqrt \gamma - \sqrt {\hat{\gamma }} )} d\rho \\ \end{aligned} $$

The derivation process of $\varepsilon (t)$ is expressed as follows:

$$ IF\;\left\{ \begin{gathered} \varpi_{1} (t)\;is\;{\kern 1pt} \beta_{i1} ,and \ldots ,and\;\varpi_{\sigma } (t)\;is\;\beta_{i\sigma } \hfill \\ \Delta_{1} (t){\kern 1pt} \;is{\kern 1pt} \;\beta_{w1} ,and \ldots and\;\Delta_{\upsilon } (t)\;is\;\beta_{w\upsilon } \hfill \\ \end{gathered} \right.,\;THEN: $$

18 $$ \begin{aligned} \varepsilon (t) & = \Sigma_{2} [\Phi (D_{i0} \Xi_{i0} \varsigma (t) + D_{i2} \Xi_{i2} x_{2} (t)) - \hat{\Phi }(D_{i0} \Xi_{i0} \hat{\varsigma }(t) + D_{i2} \Xi_{i2} \hat{x}_{2} (t)) \\ & \quad + \hat{\Phi }(D_{i0} \Xi_{i0} \varsigma (t) + D_{i2} \Xi_{i2} x_{2} (t)) - \hat{\Phi }(D_{i0} \Xi_{i0} \varsigma (t) + D_{i2} \Xi_{i2} x_{2} (t))] \\ & = \hat{\Phi }\Sigma_{2} (D_{i0} \Xi_{i0} e_{\varsigma } (t) + D_{i2} \Xi_{i2} e_{2} (t)){ + }\hat{\Phi }\Sigma_{2} (D_{i0} \Xi_{i0} \varsigma (t) \\ & \quad + D_{i2} \Xi_{i2} x_{2} (t))(\Phi /\hat{\Phi } - 1) \\ \end{aligned} $$

On the basis of (19), error function (18) is derived as (20):

19 $$ 1/\hat{\Phi } - 1/\Phi = \lambda_{1} (\left\| {\hat{V}(t)} \right\| - \left\| {V^{T} (t)} \right\|) $$

20 $$ \begin{aligned} \varepsilon (t) & = \Lambda_{(i,w)} (\varpi ,\Delta )[\hat{\Phi }\Sigma_{2} (D_{i0} \Xi_{i0} e_{\varsigma } (t) + D_{i2} \Xi_{i2} e_{2} (t)) \\ & \quad + \hat{\Phi }\Sigma_{2} (D_{i0} \Xi_{i0} \varsigma (t) + D_{i2} \Xi_{i2} x_{2} (t))\Phi \lambda_{1} (\left\| {\hat{V}(t)} \right\| - \left\| {V^{T} (t)} \right\|)] \\ \end{aligned} $$

By combining (16) and (17), the full-order observer error system is established as follows:

21 $$ \begin{aligned} \dot{e}_{\varsigma } (t) & = \dot{\varsigma } - \dot{\hat{\varsigma }} \\ & = \Lambda_{(i,w)} (\varpi ,\Delta )\{ [A_{iw0} e_{\varsigma } (t) + A_{id0} e_{\varsigma } (t - \tau (t)) + N_{i0} F_{com} (t) \\ & \quad - L_{i3} \Sigma_{2} D_{i0} \Xi_{i0} e_{\varsigma } - L_{i3} \Sigma_{2} D_{i2} \Xi_{i2} e_{2} ] + L_{i3} \Sigma_{2} V\Phi \lambda_{1} (\left\| V \right\| - \left\| {\hat{V}} \right\|)\} \\ \end{aligned} $$

where $$L_{i3} = \hat{\Phi }K_{i1}$$ and $$\Sigma_{2} = \int_{a}^{b} {C(\rho )d\rho }$$.

In the estimation process, the observer is constructed to eliminate the disturbance and estimate the faults as follows:

22 $$ \begin{aligned} & \dot{\hat{x}}_{1} (t) = \Lambda_{(i,w)} (\varpi ,\Delta )[A_{i1} \hat{x}_{1} (t) + A_{id1} \hat{x}_{1} (t - \tau (t)) \\ & \quad \quad \quad + B_{i1} u(t) + N_{i1} \hat{F}_{com} (t) + B_{id1} T_{w} \hat{\omega }(t) + K_{i2} \varepsilon (t)] \\ & \hat{x}_{2} (t) = \Lambda_{(i,w)} (\varpi ,\Delta )[ - A_{id2} \hat{x}_{2} (t - \tau (t)) - B_{i2} u(t) - N_{i2} \hat{F}_{com} (t) - B_{id2} T_{w} \hat{\omega }(t)] \\ & \hat{V}(t) = \Lambda_{(i,w)} (\varpi ,\Delta )(D_{i1} \Xi_{i1} \hat{x}_{1} (t) + D_{i2} \Xi_{i2} \hat{x}_{2} (t)) \\ & \hat{\gamma }(\rho ,u(t)) = \hat{\Phi }^{2} (C(\rho )\hat{V}(t))^{2} \\ & \dot{\hat{F}}_{com} (t) = \Lambda_{(i,w)} (\varpi ,\Delta )( - prey\{ \Gamma_{i1} \} \hat{F}_{com} (t) + prey\{ \Gamma_{i2} \} \varepsilon (t)) \\ \end{aligned} $$

where *prey*{·} means the prey adaptive strategy designed for different fault amplitudes and does not affect the derivation. The estimation error dynamics can be expressed as (24) by setting (23):

23 $$ \left\{ \begin{gathered} \tilde{F}_{com} (t) = F_{com} (t) - \hat{F}_{com} (t) \hfill \\ e_{1} = x_{1} - \hat{x}_{1} \hfill \\ \end{gathered} \right. $$

24 $$ \begin{aligned} \dot{e}_{1} (t) & = \Lambda_{(i,w)} (\varpi ,\Delta )[A_{i1} e_{1} (t) + A_{id1} e_{1} (t - \tau (t)) + N_{i1} \tilde{F}_{com} (t) + B_{id1} T_{w} \tilde{\omega }(t) \\ & \quad - L_{i4} \Sigma_{2} D_{i1} \Xi_{i1} e_{1} - L_{i4} \Sigma_{2} D_{i2} \Xi_{i2} e_{2} + L_{i4} \Sigma_{2} V\Phi \lambda_{1} (\left\| V \right\| - \left\| {\hat{V}} \right\|)] \\ \end{aligned} $$

where $L_{i4} = \hat{\Phi }K_{i2}$. Thence the fault estimation dynamic is as follows:

25 $$ \begin{aligned} \dot{\hat{F}}_{com} (t) & = \Lambda_{(i,w)} (\varpi ,\Delta )[ - prey\{ \Gamma_{i1} \} \hat{F}_{com} (t) + L_{i5} \Sigma_{2} D_{i1} \Xi_{i1} e_{1} \\ & \quad + L_{i5} \Sigma_{2} D_{i2} \Xi_{i2} e_{2} - L_{i5} \Sigma_{2} V\Phi \lambda_{1} (\left\| V \right\| - \left\| {\hat{V}} \right\|)] \\ \end{aligned} $$

and $L_{i5} = \hat{\Phi }prey\{ \Gamma_{i2} \}$. The following equation can be derived by combining systems (21), (24) and (25):

26 $$ \begin{aligned} \left[ {\begin{array}{*{20}c} {\dot{e}_{\varsigma } (t)} \\ {\dot{e}_{1} (t)} \\ {\dot{\hat{F}}_{com} (t)} \\ \end{array} } \right] & = \Lambda_{(i,w)} (\varpi ,\Delta )\left[ {\overline{A}_{iw} \left[ {\begin{array}{*{20}c} {e_{\varsigma } (t)} \\ {e_{1} (t)} \\ {\hat{F}_{com} (t)} \\ \end{array} } \right] + \left[ {\begin{array}{*{20}c} {A_{id0} e_{\varsigma } (t - \tau (t))} \\ {A_{id1} e_{1} (t - \tau (t))} \\ 0 \\ \end{array} } \right]} \right. \\ & \quad \left. { + \left[ {\begin{array}{*{20}c} {N_{i0} F_{com} (t)} \\ {N_{i1} F_{com} (t)} \\ 0 \\ \end{array} } \right] + \left[ {\begin{array}{*{20}c} { - L_{i3} \Sigma_{2} D_{i2} \Xi_{i2} e_{2} } \\ { - L_{i4} \Sigma_{2} D_{i2} \Xi_{i2} e_{2} } \\ {L_{i5} \Sigma_{2} D_{i2} \Xi_{i2} e_{2} } \\ \end{array} } \right] + \left[ {\begin{array}{*{20}c} {L_{i3} \Sigma_{2} h_{1} } \\ {L_{i4} \Sigma_{2} h_{2} } \\ { - L_{i5} \Sigma_{2} h_{2} } \\ \end{array} } \right]} \right] \\ \end{aligned} $$

where

$$ \begin{aligned} &\overline{A}_{iw} = \left[ {\begin{array}{*{20}c} {A_{iw0} - L_{i3} \Sigma_{2} D_{i0} \Xi_{i0} } & 0 & 0 \\ {\overline{T}_{iw} } & {A_{i1} - L_{i4} \Sigma_{2} D_{i1} \Xi_{i1} } & { - N_{i1} } \\ 0 & {L_{i5} \Sigma_{2} D_{i1} \Xi_{i1} } & { - \Gamma_{i1} } \\ \end{array} } \right],\\ &\overline{T}_{iw} = [\begin{array}{*{20}c} 0 & {B_{id1} T} \\ \end{array}_{w} ],\\ & h_{1} = V\Phi \lambda_{1} (\left\| V \right\| - \left\| {\hat{V}} \right\|),\\ & h_{2} = V\Phi \lambda_{2} (\left\| V \right\| - \left\| {\hat{V}} \right\|) \end{aligned} $$

The reference output $s_{\infty }$ is defined as follows:

27 $$ s_{\infty } = C_{1} e_{\varsigma } (t) + C_{2} e_{1} (t) + C_{3} e_{1} (t - \tau (t)) + C_{4} \tilde{F}_{com} (t) + C_{5} e_{2} (t) $$

For $\mu_{1} > 0$ and $\mu_{2} > 0$, the robust performance indicator is defined as follows:

28 $$ J_{\infty } = \left\| {s_{\infty } } \right\|^{2} - \mu_{1}^{2} \left\| {F_{com} (t)} \right\|^{2} - \mu_{2}^{2} \left\| {e_{2} (t)} \right\|^{2} - \overline{\delta }(Q_{1} ,Q_{2} ,Q_{3} ,Q_{4} ) $$

29 $$ \begin{aligned} \overline{\delta }(Q_{1} ,Q_{2} ,Q_{3} ,Q_{4} ) & = e_{\varsigma }^{T} (0)Q_{1} e_{\varsigma } (0) + e_{1}^{T} (0)Q_{2} e_{1} (0) \\ & \quad + \int_{ - \tau (t)}^{0} {\phi_{1}^{T} Q_{3} \phi_{1} d\alpha } + \int_{ - \tau (t)}^{0} {\phi_{2}^{T} Q_{4} \phi_{2} d\alpha } \\ \end{aligned} $$

When $$t \in [ - \tau (t),0]$$, $$\hat{\varsigma } = 0$$, $$\hat{x}_{1} = 0$$, $$\varsigma = \varphi_{1} (t)$$ and $$x_{1} = \varphi_{2} (t)$$.

#### *Theorem 1*

For matrices $C_{k} > 0$ (*k* = 1, …, 5) and $Q_{{\overline{k}}} > 0$ ($\overline{k}$ = 1, …, 4), if matrices *P*_1_, *P*_2_, *R*_1_, *R*_2_ and *L*_*i*3_ exist, then $L_{i4}$, $L_{i5}$, $prey\{ \Gamma_{i1} ,\Gamma_{i2} \}$, $\mu_{1}$ and $\mu_{2}$ ensure that (30) holds:

30 $$ \Pi = \left[ {\begin{array}{*{20}c} {\Pi_{1} } & {\Pi_{2} } \\ {\Pi_{2}^{T} } & {\Pi_{3} } \\ \end{array} } \right] + \Pi_{4}^{T} \Pi_{4} < 0 $$

where

31 $$ \Pi_{4} = [\begin{array}{*{20}c} {C_{1} } & 0 & {C_{2} } & {C_{3} } & 0 & 0 & {C_{4} } & { - C_{4} } & {C_{5} } \\ \end{array} ] $$

32 $$ \Pi_{1} = \left[ {\begin{array}{*{20}c} {\xi_{iw}^{11} } & {P_{1} A_{id0} } & {\overline{T}_{iw}^{T} P_{2}^{T} } & 0 & {P_{1} L_{i3} \Sigma_{2} } \\ * & { - R_{1} } & 0 & 0 & 0 \\ * & * & {\xi_{i}^{33} } & {P_{2} A_{id1} } & 0 \\ * & * & * & { - R_{2} } & 0 \\ * & * & * & * & { - I} \\ \end{array} } \right] $$

33 $$ \Pi_{2} = \left[ {\begin{array}{*{20}c} 0 & {P_{1} N_{i0} } & 0 & { - P_{1} L_{i3} \Sigma_{2} D_{i2} \Xi_{i2} } \\ 0 & 0 & 0 & 0 \\ {P_{2} L_{i4} \Sigma_{2} } & {P_{2} N_{i1} } & {\xi_{i}^{38} } & { - P_{2} L_{i4} \Sigma_{2} D_{i2} \Xi_{i2} } \\ 0 & 0 & 0 & 0 \\ 0 & 0 & 0 & 0 \\ \end{array} } \right] $$

34 $$ \Pi_{3} = \left[ {\begin{array}{*{20}c} { - I} & 0 & {\Sigma_{2}^{T} L_{i5} } & 0 \\ * & { - \mu_{1}^{2} } & 0 & 0 \\ * & * & { - 2\Gamma_{i1} } & {L_{5} \Sigma_{2} D_{i2} } \\ * & * & * & { - \mu_{2}^{2} } \\ \end{array} } \right] $$

35 $$ \xi_{iw}^{11} = (A_{iw0} - L_{i3} \Sigma_{2} D_{i0} \Xi_{i0} )^{T} P_{1} + P_{1} (A_{iw0} - L_{i3} \Sigma_{2} D_{i0} \Xi_{i0} ) + R_{1} + m_{1} I $$

36 $$ \xi_{i}^{33} = (A_{i1} - L_{i4} \Sigma_{2} D_{i1} \Xi_{i1} )^{T} P_{2} + P_{2} (A_{i1} - L_{i4} \Sigma_{2} D_{i1} \Xi_{i1} ) + R_{2} + m_{2} I $$

37 $$ \xi_{i}^{38} = (D_{i1}^{{}} \Xi_{i1} )^{T} \Sigma_{2}^{T} L_{i5}^{T} - P_{2} N_{i1} $$

If the inequalities $P_{1} \le Q_{1}$, $P_{2} \le Q_{2}$, $R_{1} \le Q_{3}$ and $R_{2} \le Q_{4}$ hold, then system (26) is stable and satisfies $J_{\infty } < 0$.

#### *Proof*

By selecting the positive Lyapunov functions $\chi_{1}$, $\chi_{2}$ and $\chi_{3}$:

38 $$ \chi_{1} = e_{\varsigma }^{T} P_{1} e_{\varsigma } + \int_{0}^{t} {[m_{1} e_{\varsigma }^{T} (\alpha )e_{\varsigma } (\alpha ) - h_{1}^{T} h_{1} ]d\alpha } + \int_{t - \tau (t)}^{t} {e_{\varsigma }^{T} (\alpha )R_{1} e_{\varsigma } (\alpha )d\alpha } $$

39 $$ \chi_{2} = e_{1}^{T} P_{2} e_{1} + \int_{0}^{t} {[m_{2} e_{1}^{T} (\alpha )e_{1} (\alpha ) - h_{2}^{T} h_{2} ]d\alpha } + \int_{t - \tau (t)}^{t} {e_{1}^{T} (\alpha )R_{2} e_{1} (\alpha )d\alpha } $$

40 $$ \chi_{3} = \hat{F}_{com}^{T} \hat{F}_{com} $$

The corresponding derivatives of the functions can be obtained as follows:

41 $$ \begin{aligned} \dot{\chi }_{1} & = \Lambda_{(i,w)} (\varpi ,\Delta )\{ e_{\varsigma }^{T} [(A_{iw0} - L_{i3} \Sigma_{2} D_{i0} \Xi_{i0} )^{T} P_{1} + P_{1} (A_{iw0} \\ & \quad - L_{i3} \Sigma_{2} D_{i0} \Xi_{i0} ) + R_{1} + m_{1} I]e_{\varsigma } + 2e_{\varsigma }^{T} P_{1} A_{id0} e_{\varsigma } (t - \tau (t) \\ & \quad + 2e_{\varsigma }^{T} P_{1} N_{i0} F_{com} - 2e_{\varsigma }^{T} P_{1} L_{i3} \Sigma_{2} D_{i2} \Xi_{i2} e_{2} + 2e_{\varsigma }^{T} P_{1} L_{i3} \Sigma_{2} h_{1} \\ & \quad - h_{1}^{T} h_{1} - e_{\varsigma }^{T} (t - \tau (t))R_{1} e_{\varsigma } (t - \tau (t))\} \\ \end{aligned} $$

42 $$ \begin{aligned} \dot{\chi }_{2} & = \Lambda_{(i,w)} (\varpi ,\Delta )\{ e_{1}^{T} [(A_{i1} - L_{i4} \Sigma_{2} D_{i1} \Xi_{i1} )^{T} P_{2} + R_{2} + m_{2} I \\ & \quad + P_{2} (A_{i1} - L_{i4} \Sigma_{2} D_{i1} \Xi_{i1} )]e_{1} + 2e_{1}^{T} P_{2} \overline{T}_{iw} e_{\varsigma } + 2e_{1}^{T} P_{2} N_{i1} F_{com} \\ & \quad + 2e_{1}^{T} P_{2} A_{id1} e_{1} (t - \tau (t)) - 2e_{1}^{T} P_{2} L_{i4} \Sigma_{2} D_{i2} \Xi_{i2} e_{2} - 2e_{1}^{T} P_{2} N_{i1} \hat{F}_{com} \\ & \quad + 2e_{1}^{T} P_{2} L_{i4} \Sigma_{2} h_{2} - h_{2}^{T} h_{2} - e_{1}^{T} (t - \tau (t))R_{2} e_{1} (t - \tau (t))\} \\ \end{aligned} $$

43 $$ \begin{gathered} \dot{\chi }_{3} = \Lambda_{(i,w)} (\varpi ,\Delta )( - 2prey\{ \Gamma_{i1} \} \hat{F}_{com}^{T} \hat{F}_{com} + 2e_{1}^{T} \Xi_{i1}^{T} D_{i1}^{T} \Sigma_{2}^{T} L_{i5}^{T} \hat{F}_{com} \hfill \\ {\kern 1pt} {\kern 1pt} {\kern 1pt} {\kern 1pt} {\kern 1pt} {\kern 1pt} {\kern 1pt} {\kern 1pt} {\kern 1pt} {\kern 1pt} {\kern 1pt} {\kern 1pt} {\kern 1pt} {\kern 1pt} {\kern 1pt} {\kern 1pt} {\kern 1pt} {\kern 1pt} {\kern 1pt} {\kern 1pt} {\kern 1pt} {\kern 1pt} + 2\hat{F}_{com}^{T} L_{i5} \Sigma_{2} D_{i2} \Xi_{i2} e_{2} + 2\hat{F}_{com}^{T} L_{i5} \Sigma_{2} h_{2} ) \hfill \\ \end{gathered} $$

Define *χ* as follows:

44 $$ \chi = \chi_{1} + \chi_{2} + \chi_{3} $$

An auxiliary function is set as a second performance indicator, as follows:

45 $$ J_{1} = \int_{0}^{t} {(\left\| {s_{\infty } } \right\|^{2} - \mu_{1}^{2} \left\| {F_{com} (\alpha )} \right\|^{2} - \mu_{2}^{2} \left\| {e_{2} (\alpha )} \right\|^{2} } + \dot{\chi }(\alpha ))d\alpha $$

The derivation shows that

46 $$ \left\| {s_{\infty } } \right\|^{2} - \mu_{1}^{2} \left\| {F_{com} (t)} \right\|^{2} - \mu_{2}^{2} \left\| {e_{2} (t)} \right\|^{2} + \dot{\chi } = q^{T} \Pi q $$

47 $$ q^{T} = [\begin{array}{*{20}c} {e_{\varsigma } (t)} \\ \end{array} {\kern 1pt} {\kern 1pt} {\kern 1pt} {\kern 1pt} {\kern 1pt} {\kern 1pt} \begin{array}{*{20}c} {e_{\varsigma } (t - \tau (t))} \\ \end{array} {\kern 1pt} {\kern 1pt} {\kern 1pt} {\kern 1pt} {\kern 1pt} \begin{array}{*{20}c} {F_{com} (t)} \\ \end{array} {\kern 1pt} {\kern 1pt} {\kern 1pt} {\kern 1pt} {\kern 1pt} {\kern 1pt} \begin{array}{*{20}c} {e_{2} (t)} \\ \end{array} {\kern 1pt} {\kern 1pt} {\kern 1pt} {\kern 1pt} {\kern 1pt} {\kern 1pt} \begin{array}{*{20}c} {h_{1} (t)} \\ \end{array} {\kern 1pt} {\kern 1pt} {\kern 1pt} {\kern 1pt} {\kern 1pt} {\kern 1pt} \begin{array}{*{20}c} {e_{1} (t)} \\ \end{array} {\kern 1pt} {\kern 1pt} {\kern 1pt} {\kern 1pt} {\kern 1pt} {\kern 1pt} \begin{array}{*{20}c} {e_{1} (t - \tau (t))} \\ \end{array} {\kern 1pt} {\kern 1pt} {\kern 1pt} {\kern 1pt} {\kern 1pt} \begin{array}{*{20}c} {\hat{F}_{com} (t)} \\ \end{array} ] $$

$\Pi$ satisfies conditions (30) to (37). Thus, $J_{1} < 0$. By deriving from (45) and adjusting the parameters, *J*_1_ is expressed as follows:

48 $$ \begin{aligned} J_{1} & = \int_{0}^{t} {(\left\| {s_{\infty } } \right\|^{2} - \mu_{1}^{2} \left\| {F_{com} (t)} \right\|^{2} - \mu_{2}^{2} \left\| {e_{2} (t)} \right\|^{2} )} dt + \chi (t) - \chi (0) \\ & \ge \left\| {s_{\infty } } \right\|^{2} - \mu_{1}^{2} \left\| {F_{com} (t)} \right\|^{2} - \mu_{2}^{2} \left\| {e_{2} (t)} \right\|^{2} - [e_{\varsigma }^{T} (0)P_{1} e_{\varsigma } (0) \\ & \quad + e_{1}^{T} (0)P_{2} e_{1} (0) + \int_{ - \tau (t)}^{0} {\phi_{1}^{T} R_{1} \phi_{1} d\alpha } + \int_{ - \tau (t)}^{0} {\phi_{2}^{T} R_{2} \phi_{2} d\alpha } ] \\ \end{aligned} $$

When $t \in [ - \tau (t),0]$, condition (49) can be satisfied as follows:

49 $$ [\begin{array}{*{20}c} {\hat{\varsigma }} & {\hat{x}_{1} } & \varsigma & {x_{1} } \\ \end{array} ] = [\begin{array}{*{20}c} 0 & 0 & {\varphi_{1} (t)} & {\varphi_{2} (t)} \\ \end{array} ] $$

The following inequality is obtained on the basis of the previously presented derivation and definition:

50 $$ \begin{aligned} & J_{\infty } < J_{1} + \overline{\delta }(P_{1} - Q_{1} ,P_{2} - Q_{2} ,R_{1} - Q_{3} ,R_{2} - Q_{4} ) \\ & \overline{\delta }(P_{1} - Q_{1} ,P_{2} - Q_{2} ,R_{1} - Q_{3} ,R_{2} - Q_{4} ) \\ & \quad = [e_{\varsigma }^{T} (0)P_{1} e_{\varsigma } (0) + e_{1}^{T} (0)P_{2} e_{1} (0) + \int_{ - \tau (t)}^{0} {\phi_{1}^{T} R_{1} \phi_{1} d\alpha } \\ & \quad \quad + \int_{ - \tau (t)}^{0} {\phi_{2}^{T} R_{2} \phi_{2} d\alpha } ] - [e_{\varsigma }^{T} (0)Q_{1} e_{\varsigma } (0) + e_{1}^{T} (0)Q_{2} e_{1} (0) \\ & \quad \quad + \int_{ - \tau (t)}^{0} {\phi_{1}^{T} Q_{3} \phi_{1} d\alpha } + \int_{ - \tau (t)}^{0} {\phi_{2}^{T} Q_{4} \phi_{2} d\alpha } ] \\ \end{aligned} $$

From (29), we can deduce that $J_{1} < 0$:

51 $$ \overline{\delta }(P_{1} - Q_{1} ,P_{2} - Q_{2} ,R_{1} - Q_{3} ,R_{2} - Q_{4} ) < 0 $$

Hence, $J_{\infty } < 0$, the robust stable is proved. The system can mask disturbance and accurately estimate the faults.□

### Adaptive fault tolerance

Given the estimated values, fault-tolerant controller ensures that the PDF tracking errors meet the upper bound and the angles match their ideal values. The expected output PDFs are set as follows:

52 $$ \gamma_{g} (\rho ) = \Phi_{g}^{2} (C(\rho )V_{g} )^{2} = (C(\rho )V_{g} )^{2} /(V_{g}^{T} \Sigma_{1} V_{g} ),{\kern 1pt} {\kern 1pt} {\kern 1pt} {\kern 1pt} \forall \rho \in [a,b] $$

where $\gamma_{g} (\rho )$ is the expected output PDF and $V_{g}$ is the weight corresponding to the expected output PDF. Thereafter, let

53 $$ \begin{aligned} & e_{2} = V - V_{g} \\ & \Lambda_{(i,w)} (\varpi ,\Delta )E_{i} \Xi_{i}^{ - 1} D_{i}^{ - 1} \dot{e}_{2} = \Lambda_{(i,w)} (\varpi ,\Delta )[E_{i} \Xi_{i}^{ - 1} D_{i}^{ - 1} (\dot{V} - \dot{V}_{g} )] \\ & = \Lambda_{(i,w)} (\varpi ,\Delta )E_{i} \dot{e}_{m} \\ \end{aligned} $$

The tracking error dynamics system is described as follows:

54 $$ \begin{aligned} \Lambda_{(i,w)} (\varpi ,\Delta )E_{i} \dot{e}_{m} & = \Lambda_{(i,w)} (\varpi ,\Delta )[A_{i} e_{m} + A_{id} e_{m} (t - \tau (t)) + B_{i} u(t) \\ & \quad + N_{i} F_{com} (t) + B_{id} d(t) + (A_{i} + A_{id} )x_{g} ] \\ \end{aligned} $$

where *e*_*m*_ = *x*(*t*)*-x*_*g*_. We assume that the system is regular pulseless and $$\overline{E}_{i} = L_{i1} E_{i} L_{i2} = \left[ {\begin{array}{*{20}c} {I_{q} } & 0 \\ 0 & 0 \\ \end{array} } \right]$$ and $$L_{i2}^{ - 1} e_{m} = \left[ {\begin{array}{*{20}c} {\zeta_{1} } \\ {\zeta_{2} } \\ \end{array} } \right] = \zeta$$, (54) can be rewritten as follows:

55 $$ \begin{aligned} & \Lambda_{(i,w)} (\varpi ,\Delta )\overline{E}_{i} \dot{\zeta }(t) = \Lambda_{(i,w)} (\varpi ,\Delta )[L_{1} A_{i} L_{2} \zeta (t) \\ & \quad + L_{1} A_{id} L_{2} \zeta (t - \tau (t)) + L_{1} B_{i} u(t) + L_{1} N_{i} F_{com} (t) \\ & \quad + L_{1} B_{id} d(t) + L_{1} (A_{i} + A_{id} )x_{g} \\ & \Lambda_{(i,w)} (\varpi ,\Delta )B_{i} U(t) = \Lambda_{(i,w)} (\varpi ,\Delta )[B_{i} u(t) + (A_{i} + A_{id} )x_{g} ] \\ & U(t) = u(t) + \Lambda_{(i,w)} (\varpi ,\Delta )[(B_{i}^{T} B_{i} )^{ - 1} B_{i}^{T} (A_{i} + A_{id} )x_{g} ] \\ \end{aligned} $$

For eliminating the disturbance and faults, control inputs are as follows:

56 $$ \begin{aligned} U(t) & = \Lambda_{(i,w)} (\varpi ,\Delta )[\Gamma_{i3} \int_{a}^{b} {(\Phi C(\rho )V(t)} \\ & \quad - \Phi_{g} C(\rho )V_{g} )d\rho + prey\{ \Gamma_{i4} \} F_{com} (t) + prey\{ \Gamma_{i5} \} d(t)] \\ & = \Lambda_{(i,w)} (\varpi ,\Delta )[\Phi_{g} \Gamma_{i3} \Sigma_{2} D_{i} \Xi_{i} P\zeta (t) + prey\{ \Gamma_{i5} \} d(t) \\ & \quad + \Phi_{g} \Gamma_{i3} \Sigma_{2} V(t)\Phi \lambda_{3} (\left\| {V_{g} } \right\| - \left\| {V(t)} \right\|) + prey\{ \Gamma_{i4} \} F_{com} (t)] \\ & = \Lambda_{(i,w)} (\varpi ,\Delta )[L_{i6} \Sigma_{2} D_{i} \Xi_{i} P\zeta (t) + L_{i6} \Sigma_{2} h_{3} \\ & \quad + prey\{ \Gamma_{i4} \} F_{com} (t) + prey\{ \Gamma_{i5} \} d(t)] \\ \end{aligned} $$

where $$L_{i6} = \Phi_{g} \Gamma_{i3}$$ and $$h_{3} = \Phi \lambda_{3} (\left\| {V_{g} } \right\| - \left\| {V(t)} \right\|)V(t)$$.

#### *Theorem 2*

If a positive definite matrix *P* exists and matrices Γ_*i*3_, *prey*{Γ_*i*4_} and *prey*{Γ_*i*5_} ensure that the LMI in (57) and (58) is valid, then system (54) is stable.

57 $$ \Pi_{8} = \left[ {\begin{array}{*{20}c} {\Upsilon_{i4} } & {A_{id} P^{T} } & {B_{i} L_{i6} \Sigma_{2} L_{2}^{ - 1} P^{T} } & {B_{i} \Gamma_{i4} /\eta_{2} } & {B_{i} \Gamma_{i5} /\eta_{4} } \\ * & { - R_{1} } & 0 & 0 & 0 \\ * & * & { - I} & 0 & 0 \\ * & * & * & { - I} & 0 \\ * & * & * & * & { - I} \\ \end{array} } \right] < 0 $$

58 $$ \begin{gathered} \Upsilon_{i4} = A_{i} P^{T} + PA_{i}^{T} + PL_{2}^{ - T} R_{1} L_{2}^{ - 1} P^{T} + 2B_{i} L_{i6} \Sigma_{2} D_{i} \Xi_{i} P^{T} \hfill \\ {\kern 1pt} {\kern 1pt} {\kern 1pt} {\kern 1pt} {\kern 1pt} {\kern 1pt} {\kern 1pt} {\kern 1pt} {\kern 1pt} {\kern 1pt} {\kern 1pt} {\kern 1pt} {\kern 1pt} {\kern 1pt} {\kern 1pt} {\kern 1pt} {\kern 1pt} {\kern 1pt} {\kern 1pt} {\kern 1pt} {\kern 1pt} {\kern 1pt} {\kern 1pt} {\kern 1pt} {\kern 1pt} {\kern 1pt} + PL_{2}^{ - T} \alpha_{3} L_{2}^{ - 1} P^{T} + N_{i} N_{i}^{T} /\eta_{1}^{2} + B_{id} B_{id}^{T} /\eta_{3}^{2} + \lambda I \hfill \\ \end{gathered} $$

#### *Proof*

By selecting the following Lyapunov function:

59 $$ \nu = \Lambda_{(i,w)} (\varpi ,\Delta )\zeta^{T} \overline{E}_{i}^{T} \overline{P}^{ - T} \zeta + \int_{t - \tau (t)}^{t} {\zeta^{T} (s)R_{1} \zeta (s)ds} + \int_{0}^{t} {\alpha_{3} \zeta^{T} \zeta } ds - \int_{0}^{t} {h_{3}^{T} h_{3} } ds $$

Then the derivative of $\nu$ is obtained as follows:

60 $$ \begin{aligned} \dot{\nu } & = \Lambda_{(i,w)} (\varpi ,\Delta )\{ \zeta^{T} [\overline{P}^{ - 1} L_{1} A_{i} L_{2} + 2\overline{P}^{ - 1} \nabla + R_{1} + L_{2}^{T} A_{i}^{T} L_{1}^{T} \overline{P}^{ - T} ]\zeta \\ & \quad + 2\zeta^{T} \overline{P}^{ - 1} L_{1} A_{id} L_{2} \zeta (t - \tau (t)) + 2\zeta^{T} \overline{P}^{ - 1} L_{1} B_{id} d(t) + 2\zeta^{T} (\overline{P}^{ - 1} L_{1} N_{i} \\ & \quad + \overline{P}^{ - 1} L_{1} B_{i} prey\{ \Gamma_{i4} \} )F_{com} (t) + 2\zeta^{T} \overline{P}^{ - 1} L_{1} B_{i} prey\{ \Gamma_{i5} \} d(t) \\ & \quad - \zeta^{T} (t - \tau (t))R_{1} \zeta (t - \tau (t)) + 2\zeta^{T} \overline{P}^{ - 1} L_{1} B_{i} L_{i6} \Sigma_{2} h_{3} + \alpha_{3} \zeta^{T} \zeta - h_{3}^{T} h_{3} \} \\ \end{aligned} $$

Inequality (61) can be expressed as follows^29^:

61 $$ h_{3}^{T} h_{3} \le \left( {\lambda_{3} \left\| {D_{i} \Xi_{i} L_{2} } \right\|/\sqrt {\left\| {\Sigma_{1} } \right\|} } \right)^{2} \zeta^{T} (t)\zeta (t) $$

By letting $\alpha_{3} = \left( {\lambda_{3} \left\| {D_{i} \Xi_{i} L_{2} } \right\|/\sqrt {\left\| {\Sigma_{1} } \right\|} } \right)^{2}$, $\nu \ge 0$ is ensured to be constantly right, hence

62 $$ \begin{aligned} & \dot{\nu } \le \Lambda_{(i,w)} (\varpi ,\Delta )\{ \zeta^{T} [\overline{P}^{ - 1} L_{1} A_{i} L_{2} + L_{2}^{T} A_{i}^{T} L_{1}^{T} \overline{P}^{ - T} + 2\overline{P}^{ - 1} \nabla + \alpha_{3} \\ & \quad + R_{1} ]\zeta + \eta_{4}^{2} d^{T} (t)d(t) + 2\zeta^{T} \overline{P}^{ - 1} L_{1} A_{id} L_{2} \zeta (t - \tau (t)) - h_{3}^{T} h_{3} \\ & \quad { + }\eta_{3}^{2} d(t)d(t) + \eta_{1}^{2} F_{com}^{T} (t)F_{com} (t) - \zeta^{T} (t - \tau (t))R_{1} \zeta (t - \tau (t)) \\ & \quad + 2\zeta^{T} \overline{P}^{ - 1} L_{1} B_{i} L_{i6} \Sigma_{2} h_{3} + \eta_{2}^{2} F_{com}^{T} (t)F_{com} (t) \\ & \quad + \zeta^{T} \overline{P}^{ - 1} L_{1} B_{i} prey\{ \Gamma_{i5} \} (\overline{P}^{ - 1} L_{1} B_{i} prey\{ \Gamma_{i5} \} )^{T} \zeta \\ & \quad + \zeta^{T} \overline{P}^{ - 1} L_{1} N_{i} (\overline{P}^{ - 1} L_{1} N_{i} )^{T} \zeta /\eta_{1}^{2} + \zeta^{T} \overline{P}^{ - 1} L_{1} B_{id} (\overline{P}^{ - 1} L_{1} B_{id} )^{T} \zeta /\eta_{3}^{2} \\ & \quad + \zeta^{T} \overline{P}^{ - 1} L_{1} B_{i} prey\{ \Gamma_{i4} \} (\overline{P}^{ - 1} L_{1} B_{i} prey\{ \Gamma_{i4} \} )^{T} \zeta /\eta_{2}^{2} \\ & \le q^{T} \Pi_{5} q + (\eta_{1}^{2} + \eta_{2}^{2} )F_{com}^{T} (t)F_{com} (t) + (\eta_{3}^{2} + \eta_{4}^{2} )d^{T} (t)d(t) \\ \end{aligned} $$

where

63 $$ \nabla { = }L_{1} B_{i} L_{i6} \Sigma_{2} D_{i} \Xi_{i} L_{2} $$

64 $$ q^{T} = \begin{array}{*{20}c} {[\zeta^{T} } & {\zeta^{T} (t - \tau (t))} & {h_{3}^{T} } \\ \end{array} ] $$

65 $$ \Pi_{5} = \Lambda_{(i,w)} (\varpi ,\Delta )\left[ {\begin{array}{*{20}c} {\Upsilon_{i1} } & {\overline{P}^{ - 1} L_{1} A_{id} L_{2} } & {\overline{P}^{ - 1} L_{1} B_{i} L_{i6} \Sigma_{2} } \\ * & { - R_{1} } & 0 \\ * & * & { - I} \\ \end{array} } \right] $$

66 $$ \begin{aligned} \Upsilon_{i1} & = \overline{P}^{ - 1} L_{1} A_{i} L_{2} + L_{2}^{T} A_{i}^{T} L_{1}^{T} \overline{P}^{ - T} + R_{1} + 2\overline{P}^{ - 1} \nabla + \alpha_{3} \\ & \quad + \overline{P}^{ - 1} L_{1} N_{i} (\overline{P}^{ - 1} L_{1} N_{i} )^{T} /\eta_{1}^{2} + \overline{P}^{ - 1} L_{1} B_{id} (\overline{P}^{ - 1} L_{1} B_{id} )^{T} /\eta_{3}^{2} \\ & \quad + \overline{P}^{ - 1} L_{1} B_{i} prey\{ \Gamma_{i4} \} (\overline{P}^{ - 1} L_{1} B_{i} prey\{ \Gamma_{i4} \} )^{T} /\eta_{2}^{2} \\ & \quad + \overline{P}^{ - 1} L_{1} B_{i} prey\{ \Gamma_{i5} \} (\overline{P}^{ - 1} L_{1} B_{i} prey\{ \Gamma_{i5} \} )^{T} /\eta_{4}^{2} \\ \end{aligned} $$

By substituting $\overline{P} = L_{1} PL_{2}^{ - T}$ and $\overline{P}^{ - 1} = L_{2}^{T} P^{ - 1} L_{1}^{ - 1}$ into $\Pi_{5}$, the following equations can be derived as

67 $$ \Pi_{6} = \Lambda_{(i,w)} (\varpi ,\Delta )\left[ {\begin{array}{*{20}c} {\Upsilon_{i2} } & {L_{2}^{T} P^{ - 1} A_{id} L_{2} } & {L_{2}^{T} P^{ - 1} B_{i} L_{i6} \Sigma_{2} } \\ * & { - R_{1} } & 0 \\ * & * & { - I} \\ \end{array} } \right] $$

68 $$ \begin{aligned} \Upsilon_{i2} & = L_{2}^{T} P^{ - 1} A_{i} L_{2} + L_{2}^{T} A_{i}^{T} P^{ - T} L_{2} + R_{1} + 2L_{2}^{T} P^{ - 1} B_{i} L_{i6} \Sigma_{2} D_{i} \Xi_{i} L_{2} \\ & \quad + L_{2}^{T} P^{ - 1} N_{i} N_{i}^{T} P^{ - T} L_{2} /\eta_{1}^{2} + L_{2}^{T} P^{ - 1} B_{id} B_{id}^{T} P^{ - T} L_{2} /\eta_{3}^{2} + \alpha_{3} \\ & \quad + L_{2}^{T} P^{ - 1} B_{i} prey\{ \Gamma_{i4} \Gamma_{i4}^{T} \} B_{i}^{T} P^{ - T} L_{2} /\eta_{2}^{2} \\ & \quad + L_{2}^{T} P^{ - 1} B_{i} prey\{ \Gamma_{i5} \Gamma_{i5}^{T} \} B_{i}^{T} P^{ - T} L_{2} /\eta_{4}^{2} \\ \end{aligned} $$

Π_6_ is multiplied by $$diag(\begin{array}{*{20}c} {PL_{2}^{ - T} } & I & I \\ \end{array} )$$ on the left and $$diag(\begin{array}{*{20}c} {L_{2}^{ - 1} P^{T} } & I & I \\ \end{array} )$$ on the right. Then first row first column element of Π_6_ is added by $\lambda I$. Hence, (69) is obtained, where $\lambda$ is a small positive constant.

69 $$ \Pi_{7} = \Lambda_{(i,w)} (\varpi ,\Delta )\left[ {\begin{array}{*{20}c} {\Upsilon_{i3} } & {A_{id} P^{T} } & {B_{i} L_{i6} \Sigma_{2} L_{2}^{ - 1} P^{T} } \\ * & { - R_{1} } & 0 \\ * & * & { - I} \\ \end{array} } \right] $$

70 $$ \begin{aligned} \Upsilon_{i3} & = A_{i} P^{T} + PA_{i}^{T} + PL_{2}^{ - T} R_{1} L_{2}^{ - 1} P^{T} + 2B_{i} L_{i6} \Sigma_{2} D_{i} \Xi_{i} P^{T} { + }\lambda I \\ & \quad + PL_{2}^{ - T} \alpha_{3} L_{2}^{ - 1} P^{T} + N_{i} N_{i}^{T} /\eta_{1}^{2} + B_{id} B_{id}^{T} /\eta_{3}^{2} \\ & \quad + B_{i} prey\{ \Gamma_{i4} \Gamma_{i4}^{T} \} B_{i}^{T} /\eta_{2}^{2} + B_{i} prey\{ \Gamma_{i5} \Gamma_{i5}^{T} \} B_{i}^{T} /\eta_{4}^{2} \\ \end{aligned} $$

When $\Pi_{8} < 0$, the following inequality is obtained:

71 $$ \dot{\nu } \le - \lambda \zeta^{T} \zeta + (\eta_{1}^{2} + \eta_{2}^{2} )F_{com}^{T} (t)F_{com} (t) + (\eta_{3}^{2} + \eta_{4}^{2} )d^{T} (t)d(t) $$

We obtain $\Pi_{7} < 0$ and $\Pi_{8} < 0$, which are equivalent, based on Schur complement theory. Therefore, when $\Pi_{8} < 0$ and

72 $$ \left\| \zeta \right\|^{2} > \frac{{M_{f}^{2} (\eta_{1}^{2} + \eta_{2}^{2} ) + M_{d}^{2} (\eta_{3}^{2} + \eta_{4}^{2} )}}{\lambda } $$

$\dot{\nu } < 0$ is confirmed. Hence, the error system is stable.□

In the input (56), the actual value is replaced with the fault estimated value. Thus, the fault-tolerant tracking controller can be expressed as follows:

73 $$ \begin{aligned} u(t) & = \Lambda_{(i,w)} (\varpi ,\Delta )[U(t) - (B_{i}^{T} B_{i} )^{ - 1} B_{i}^{T} (A_{i} + A_{id} )x_{g} ] \\ & = \Lambda_{(i,w)} (\varpi ,\Delta )[L_{i6} \Sigma_{2} D_{i} \Xi_{i} P\zeta (t) - (B_{i}^{T} B_{i} )^{ - 1} B_{i}^{T} (A_{i} + A_{id} )x_{g} \\ & \quad + prey\{ \Gamma_{i5} \} \hat{d}(t) + prey\{ \Gamma_{i4} \} \hat{F}_{com} (t) + L_{i6} \Sigma_{2} h_{3} ] \\ \end{aligned} $$

### Bionic parameter varying architecture

**Figure 3:**
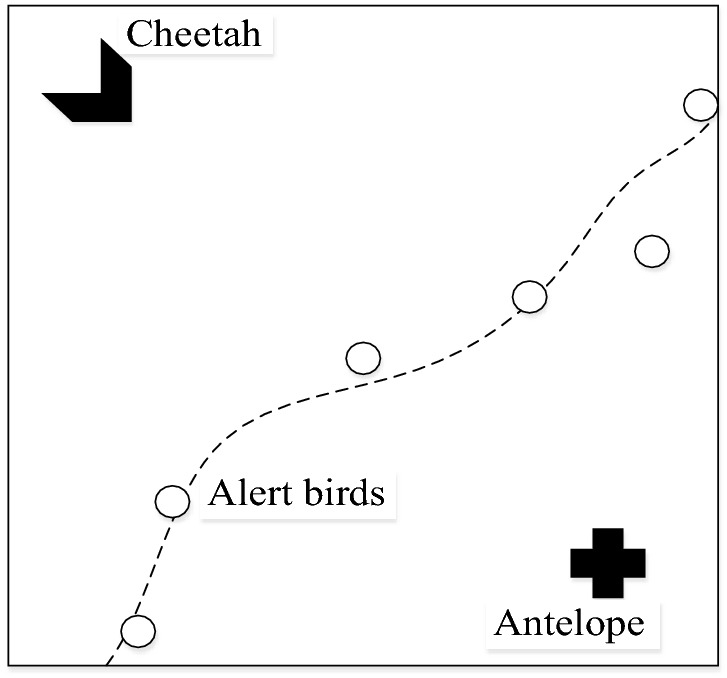
Positional relationship of two animals.

A bionic strategy that mimics animal predation allows parameters set to be optimal for incipient actuator faults, enhancing the tracking performance. An antelope can use the surrounding birds to judge threat from cheetah and respond to it by running or continue grazing^2^. Figure 3 shows the position.

Prey strategy originates from the cheetah/antelope hunting process. Accordingly, we replace cheetah and antelope with fault and controller, respectively. Tables 1 and 2 provides the corresponding relationship of insensitive prey strategy, where Γ_*i*11_, Γ_*i*21_, Γ_*i*41_, Γ_*i*51_, Γ_*i*12_, Γ_*i*22_, Γ_*i*42_, Γ_*i*52_ are the LMI-compliant adaptive learning rates, $\kappa \in R^{ + }$ is approximately zero.

**Table 1:** Insensitive prey strategy.

Cheetah		Antelope	
Distance	Status	Judgment	Response
Far	Progressive	Safe	Static
Close	Progressive	Safe	Static
Close	Run	Threat	Run

**Table 2:** Insensitive prey algorithm of fault-tolerant control.

Fault		Controller	
Distance	Status	Judgment	Response
(0, *κ*]	Fault free	Incipient impact	Γ_*i*11_, Γ_*i*21_, Γ_*i*41_, Γ_*i*51_
(*κ*, 0.1‖*u*(*t*)‖)	Fault *F*_1_(*t*)	Incipient impact	Γ_*i*11_, Γ_*i*21_, Γ_*i*41_, Γ_*i*51_
(0.1‖*u*(*t*)‖, *Mf*)	Fault*F*(*t*)	Big impact	Γ_*i*12_, Γ_*i*22_, Γ_*i*42_, Γ_*i*52_

#### *Remark 3*

The boundary state cheetah (progressive, close) corresponds to the boundary state interval $(\kappa ,{\kern 1pt} {\kern 1pt} {\kern 1pt} 0.1\left\| {u(t)} \right\|)$ in which *u*(*t*) determines the fault magnitude in Tables 1 and 2, $\kappa \ll 0.1\left\| {u(t)} \right\|$. The insensitive prey controller determines that the faults in the interval have minimal effect, thereby retaining the learning rates. The sensitive prey controller is the opposite.

**Figure 4:**
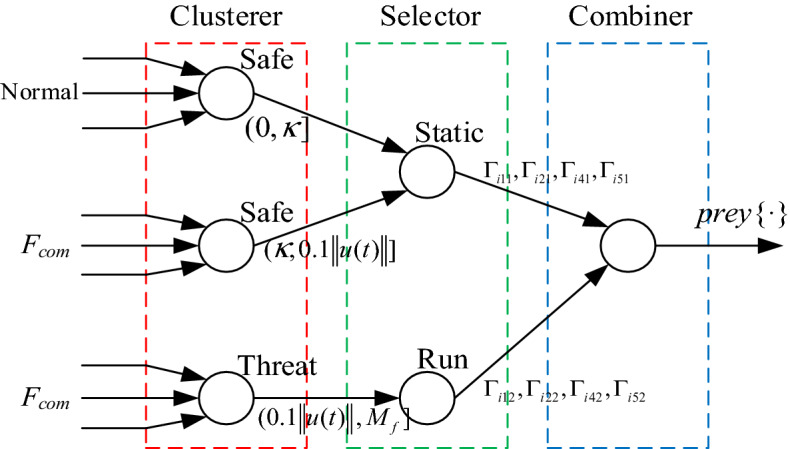
Strategic architecture.

Figure 4 is a decision tree showing the architecture of the prey algorithm. The algorithm steps are as follows:

*Step 1* Fault amplitude is less than or equal to *κ*, no fault, parameters are set to Γ_*i*11_/Γ_*i*21_/Γ_*i*41_/Γ_*i*51_.

*Step 2* Fault amplitude is larger than *κ* and less than or equal to $0.1\left\| {u(t)} \right\|$, incipient fault, parameters are insensitive and set to Γ_*i*11_/Γ_*i*21_/Γ_*i*41_/Γ_*i*51_.

*Step 3* Fault amplitude is larger than $0.1\left\| {u(t)} \right\|$, large value fault, parameters are set to Γ_*i*12_/Γ_*i*22_/Γ_*i*42_/Γ_*i*52_.

*Step 4* Return to Step 1 without modifying content.

On the basis of Table 1 and 2, and Fig. 2, (22) and (73) can be improved to prey adaptive estimation and fault-tolerant controller as follows:

74 $$ \left\{ \begin{gathered} \dot{\hat{F}}_{com} (t) = \Lambda_{(i,w)} (\varpi ,\Delta )( - prey\{ \Gamma_{i1} \} \hat{F}_{com} (t) + prey\{ \Gamma_{i2} \} \varepsilon (t)) \hfill \\ u(t) = \Lambda_{(i,w)} (\varpi ,\Delta )[L_{i6} \Sigma_{2} D_{i} \Xi_{i} P\zeta (t) + L_{i6} \Sigma_{2} h_{3} + prey\{ \Gamma_{i5} \} \hat{d}(t) \hfill \\ \quad \quad \quad + prey\{ \Gamma_{i4} \} \hat{F}_{com} (t) - (B_{i}^{T} B_{i} )^{ - 1} B_{i}^{T} (A_{i} + A_{id} )x_{g} ] \hfill \\ \end{gathered} \right. $$

where $prey\{ \Gamma_{i1} ,\Gamma_{i2} ,\Gamma_{i4} ,\Gamma_{i5} \}$ are the hybrid learning rates designed according to the prey strategy satisfied:

75 $$ prey\{ \Gamma_{i1} ,\Gamma_{i2} \} = \left\{ \begin{gathered} \Gamma_{i11} ,{\kern 1pt} {\kern 1pt} {\kern 1pt} {\kern 1pt} {\kern 1pt} \Gamma_{i21} ,{\kern 1pt} {\kern 1pt} {\kern 1pt} {\kern 1pt} {\kern 1pt} {\kern 1pt} {\kern 1pt} {\kern 1pt} {\kern 1pt} {\kern 1pt} {\kern 1pt} {\kern 1pt} {\kern 1pt} \left\| {\hat{F}_{com} (t)} \right\| \in (0,{\kern 1pt} {\kern 1pt} {\kern 1pt} \kappa ]{\kern 1pt} {\kern 1pt} {\kern 1pt} or{\kern 1pt} {\kern 1pt} {\kern 1pt} (\kappa ,{\kern 1pt} {\kern 1pt} {\kern 1pt} 0.1\left\| {u(t)} \right\|] \hfill \\ \Gamma_{i12} ,{\kern 1pt} {\kern 1pt} {\kern 1pt} {\kern 1pt} {\kern 1pt} \Gamma_{i22} ,{\kern 1pt} {\kern 1pt} {\kern 1pt} {\kern 1pt} {\kern 1pt} {\kern 1pt} {\kern 1pt} {\kern 1pt} {\kern 1pt} {\kern 1pt} {\kern 1pt} {\kern 1pt} \left\| {\hat{F}_{com} (t)} \right\| \in (0.1\left\| {u(t)} \right\|,{\kern 1pt} {\kern 1pt} {\kern 1pt} M_{f} ] \hfill \\ \end{gathered} \right. $$

76 $$ prey\{ \Gamma_{i4} ,\Gamma_{i5} \} = \left\{ \begin{gathered} \Gamma_{i41} ,{\kern 1pt} {\kern 1pt} {\kern 1pt} {\kern 1pt} {\kern 1pt} \Gamma_{i51} ,{\kern 1pt} {\kern 1pt} {\kern 1pt} {\kern 1pt} {\kern 1pt} {\kern 1pt} {\kern 1pt} {\kern 1pt} {\kern 1pt} {\kern 1pt} {\kern 1pt} {\kern 1pt} {\kern 1pt} \left\| {\hat{F}_{com} (t)} \right\| \in (0,{\kern 1pt} {\kern 1pt} {\kern 1pt} \kappa ]{\kern 1pt} {\kern 1pt} {\kern 1pt} or{\kern 1pt} {\kern 1pt} {\kern 1pt} (\kappa ,{\kern 1pt} {\kern 1pt} {\kern 1pt} 0.1\left\| {u(t)} \right\|] \hfill \\ \Gamma_{i42} ,{\kern 1pt} {\kern 1pt} {\kern 1pt} {\kern 1pt} {\kern 1pt} \Gamma_{i52} ,{\kern 1pt} {\kern 1pt} {\kern 1pt} {\kern 1pt} {\kern 1pt} {\kern 1pt} {\kern 1pt} {\kern 1pt} {\kern 1pt} {\kern 1pt} {\kern 1pt} {\kern 1pt} \left\| {\hat{F}_{com} (t)} \right\| \in (0.1\left\| {u(t)} \right\|,{\kern 1pt} {\kern 1pt} {\kern 1pt} M_{f} ] \hfill \\ \end{gathered} \right. $$

#### *Remark 4*

Prey algorithm makes estimation and fault-tolerant control processes more sensitive to the incipient deviation, hence HFV has the ability to predict and repair early signs. We define a set of faults with incipient deviation element and other deviation element to indicate that the controller can handle the individual occurrence of all fault deviation elements (*F*_1_(*t*) and *F*(*t*)).

## Simulation experiment

### HFV model

The validity should be verified via a semi-physical simulation. The PDFs of HFV that describes the attitude angle uncertainty are approximated by the weights and B-spline similar to that in (7) as follows:

77 $$ \begin{aligned} \varphi_{11} (\rho ) & = \varphi_{22} (\rho ) = \varphi_{33} (\rho ) = 0.8(\rho - 4)^{2} I_{3} \\ & \quad + ( - \rho^{2} + 5\rho - 23.5)I_{4} + 0.8(\rho - 7)^{2} I_{5} \\ \end{aligned} $$

78 $$ \begin{aligned} \varphi_{12} (\rho ) & = \varphi_{23} (\rho ) = \varphi_{31} (\rho ) = 0.3(\rho - 3)^{2} I_{2} \\ & \quad + ( - \rho^{2} + 8\rho - 19.5)I_{3} + 0.8(\rho - 6)^{2} I_{4} \\ \end{aligned} $$

79 $$ \begin{aligned} \varphi_{13} (\rho ) & = \varphi_{21} (\rho ) = \varphi_{32} (\rho ) = 0.7(\rho - 2)^{2} I_{1} \\ & \quad + ( - \rho^{2} + 10\rho - 11.5)I_{2} + 0.1(\rho - 5)^{2} I_{3} \\ \end{aligned} $$

where $I_{{\overline{i}}} (\overline{i} = 1,...,5)$ are interval functions defined as follows:

$$ I_{{\overline{i}}} = \left\{ {\begin{array}{*{20}l} {1,} \hfill & {\rho \in [\overline{i} + 1,\overline{i} + 2]} \hfill \\ {0,} \hfill & {otherwise} \hfill \\ \end{array} } \right. $$

Based on system (1) and the characteristics of the three outputs in HFV, the basis function matrix has three vectors generating three attitude angle PDFs. Both the reference *ρ*_in_ and the outputs *ρ*_out_ in Fig. 1 can be converted into corresponding PDFs.

In the fuzzy HFV dynamics, the prerequisite variable is assumed to be *w*_3_, which is an element of the weight vector *V*(*t*). In the fuzzy disturbance dynamics, the prerequisite variable is assumed to be *d*_1_, which is.

an element of the rudder interference *d*(*t*). The membership functions are expressed as follows:

80 $$ \left\{ \begin{gathered} h_{1} (\varpi ) = 1/[1 + \exp ( - 1.73w_{3} )] \hfill \\ h_{2} (\varpi ) = 1 - h_{1} \hfill \\ \end{gathered} \right. $$

81 $$ \left\{ \begin{gathered} h_{1} (\Delta ) = 1/[1 + \exp (6 + 28d_{3} )] \hfill \\ h_{2} (\Delta ) = 1 - h_{1} (\Delta ) \hfill \\ \end{gathered} \right. $$

Two rules of double-fuzzy approximation systems:

Rule 1: IF *w*_3_ is approximately 0.8, THEN: {*E*_1_ *A*_1_ *A*_1_*d H*_1_ *B*_1_ *D*_1_Ξ_1_ *B*_1_*d*},

Rule 2: IF *w*_3_ is approximately 0.1, THEN: {*E*_2_ *A*_2_ *A*_2_*d H*_2_ *B*_2_ *D*_2_Ξ_2_ *B*_2_*d*}.

Rule 1: IF *d*_1_ is approximately 0.04, THEN: {*T*_1_ Ω_1_},

Rule 2: IF *d*_1_ is approximately 0.12, THEN: {*T*_2_ Ω_2_}.

The inertia (t × m^2^) in (5) and (6) is expressed as follows: $$J = \left[ {\begin{array}{*{20}c} {55.486} & 0 & { - 23.002} \\ 0 & {1136.949} & 0 \\ { - 23.002} & 0 & {1376.852} \\ \end{array} } \right]$$.

The system state matrices can be calculated by setting the linearised roller speed and system (1). Hence, the parameter matrices in systems (10) and (11) are expressed as follows:

$$ \begin{gathered} E_{1} = E_{2} = \left[ {\begin{array}{*{20}c} 1 & 1 & 0 \\ 0 & 1 & 0 \\ 0 & 0 & 0 \\ \end{array} } \right],\quad \Xi_{1} = \Xi_{2} = \left[ {\begin{array}{*{20}c} {0.99} & 0 & {0.06} \\ {0.06} & 3 & { - 0.99} \\ 0 & 1 & 0 \\ \end{array} } \right],\quad A_{1} = \left[ {\begin{array}{*{20}c} {0.05} & {3.02} & { - 2.5} \\ { - 0.01} & 0 & {0.25} \\ 0 & { - 0.11} & {0.05} \\ \end{array} } \right],\quad A_{2} = \left[ {\begin{array}{*{20}c} {0.06} & {2.98} & { - 3.75} \\ { - 0.01} & 0 & {0.37} \\ {0.01} & { - 0.19} & {0.06} \\ \end{array} } \right], \hfill \\ A_{1d} = \left[ {\begin{array}{*{20}c} { - 0.1} & { - 0.5} & {0.12} \\ {0.2} & { - 0.25} & {0.06} \\ 0 & 0 & {0.6} \\ \end{array} } \right],\quad A_{2d} = \left[ {\begin{array}{*{20}c} { - 0.1} & { - 0.3} & { - 0.1} \\ 0 & { - 0.05} & { - 0.1} \\ 0 & 0 & { - 0.9} \\ \end{array} } \right],\quad B_{1} = \left[ {\begin{array}{*{20}c} 1 & 1 & { - 0.01} \\ 0 & 2 & { - 0.02} \\ {0.002} & {0.005} & {0.1} \\ \end{array} } \right],\quad B_{2} = \left[ {\begin{array}{*{20}c} {0.9} & {0.9} & { - 0.01} \\ 0 & 2 & { - 0.02} \\ {0.001} & {0.001} & {0.1} \\ \end{array} } \right], \hfill \\ D_{1} = \left[ {\begin{array}{*{20}c} 1 & 0 & 0 \\ 0 & 1 & 0 \\ 0 & 0 & 1 \\ \end{array} } \right],\quad D_{2} = \left[ {\begin{array}{*{20}c} 2 & 0 & 0 \\ 0 & 3 & 0 \\ 0 & 0 & 5 \\ \end{array} } \right],\quad H_{1} = \left[ {\begin{array}{*{20}c} {0.1} \\ {0.3} \\ {0.3} \\ \end{array} } \right]^{T} ,\quad H_{2} = \left[ {\begin{array}{*{20}c} {0.1} \\ {0.2} \\ {0.2} \\ \end{array} } \right]^{T} ,\quad B_{1d} = B_{2d} = \left[ {\begin{array}{*{20}c} {0.1} & {0.1} & { - 0.01} \\ 0 & {0.2} & { - 0.02} \\ {0.002} & {0.005} & {0.1} \\ \end{array} } \right]. \hfill \\ \end{gathered} $$

*H*_1_ and *H*_2_ are the equivalent matrices of *N*_1_ and *N*_2_. Delay time: $\tau (t) = 0.5(\sin t + 1)$, the parameter matrices of *d*(*t*):

$$T_{1} = \left[ {\begin{array}{*{20}c} 2 & 0 & 0 \\ 0 & 0 & 0 \\ 0 & 0 & 0 \\ \end{array} } \right],\quad T_{2} = \left[ {\begin{array}{*{20}c} 1 & 0 & 0 \\ 0 & 0 & 0 \\ 0 & 0 & 0 \\ \end{array} } \right],\quad \Omega_{1} = \left[ {\begin{array}{*{20}c} 0 & 4 & 0 \\ { - 4} & 1 & 0 \\ 0 & 0 & 2 \\ \end{array} } \right],\quad \Omega_{2} = \left[ {\begin{array}{*{20}c} 0 & 8 & 0 \\ { - 3} & 0 & 0 \\ 0 & 0 & 1 \\ \end{array} } \right].$$

The expected weight matrix is set as *V*_*g*_ = [0.7 0.7 0.79]^*T*^. According to the incipient fault *F*_1_(*t*) defined by inequality (3) and the relationship between states and weights, we set the incipient and standard deviation faults as follows:

82 $$ F_{1} (t) = \left\{ {\begin{array}{*{20}c} {0,t < 5s} \\ {0.07,t \ge 5s} \\ \end{array} } \right. $$

83 $$ F(t) = \left\{ {\begin{array}{*{20}c} {0,t < 5s} \\ {0.{25},t \ge 5s} \\ \end{array} } \right. $$

Reference input parameters are taken as *C*_4_ = *C*_5_ = 0.1, *C*_1_ = [0.1 0.1 0.1 0.1 0.1], *C*_2_ = *C*_3_ = [0.2 0.2]. This section solves the LMI in Theorems 1 and 2 for: *L*_*i*3_–*L*_*i*5_, Γ_*i*1_–Γ_*i*5_, prey learning rates Γ_*i*11_, Γ_*i*21_, Γ_*i*41_, Γ_*i*51_, Γ_*i*12_, Γ_*i*22_, Γ_*i*42_, and Γ_*i*52_ are obtained through repeated experiments. Thereafter, the ma-trices are substituted into the stochastic HFV.

### Platform and results

We use the Links-Box semi-physical simulator to verify the effectiveness of the algorithms. Links-Box automatically converts the MATLAB simulation model of HFV to the embedded control prototype and support engineering hardware to test in the model. Then HFV physical device defined in the hardware environment can dynamically verify the controller performance. The features of software package Links-RT are:

1. Adapt to the HFV model built in MATLAB;
2. Provide input/output hardware to enable users to integrate the hardware environment into HFV model;
3. Automatic conversion of MATLAB model codes to VxWorks codes;
4. Provide communication, storage, scheduling and other services in VxWorks.

**Figure 5:**
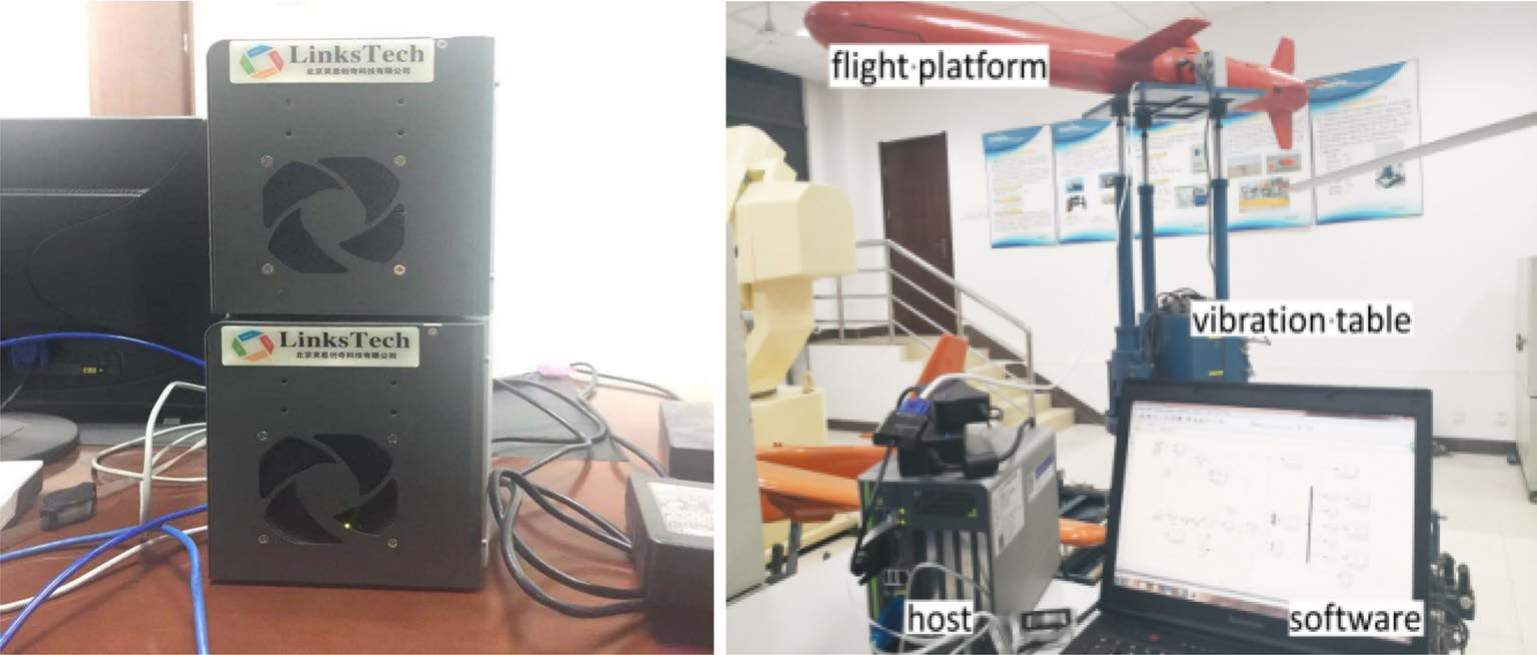
Semi-physical simulation platform.

The simulation platform is shown in Fig. 5.

Table 3 compares the results of the traditional method (TM) of HFV in^11^ and our new method (NM). e_ss1_ and e_ss2_ are the steady-state error absolute values of estimation and tolerance. Me, Mi and Ma are the mean, minimum and maximum, respectively. Table 3 shows that the method in^11^ is insufficiently effective for the HFV with non-Gaussian stochastic attitudes. The NM is more stable and accurate than TM.

**Table 3:** Comparison between TM and NM.

Error	*F*_1_(*t*)			*F*(*t*)		
	*ρ*_1_	*ρ*_2_	*ρ*_3_	*ρ*_1_	*ρ*_2_	*ρ*_3_
TM (e_ss1_)/rad	Me: 0.04	0.04	0.04	0.22	0.22	0.22
	Mi: 0.013	0.012	0.014	0.219	0.218	0.219
	Ma: 0.07	0.07	0.07	0.221	0.221	0.222
NM (e_ss1_)/rad	Me: 0	0.0001	0.0001	0.0001	0.0002	0.0001
	Mi: 0	0	0	0	0	0
	Ma: 0.0001	0.0002	0.0001	0.0001	0.0003	0.0002
TM (e_ss2_)/deg	Me: 0.09	0.08	0.08	0.07	0.06	0.07
	Mi: 0	0	0	0	0	0
	Ma: 0.14	0.12	0.13	0.11	0.1	0.11
NM (e_ss2_)/deg	Me: 0.012	0.011	0.01	0.01	0.008	0.008
	Mi: 0	0	0	0	0	0
	Ma: 0.02	0.017	0.016	0.014	0.011	0.011

**Figure 6:**
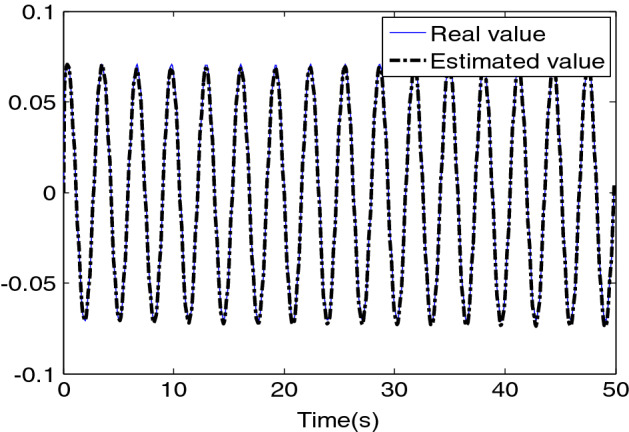
Disturbance curve and its estimation.

Augmented observer (16) shows the ideal and estimation results of disturbance in Fig. 6. The function vector is as follows:

82 $$ d(t) = [\begin{array}{*{20}c} {0.07\sin (t + 0.32)} & 0 & 0 \\ \end{array} ]^{T} $$

**Figure 7:**
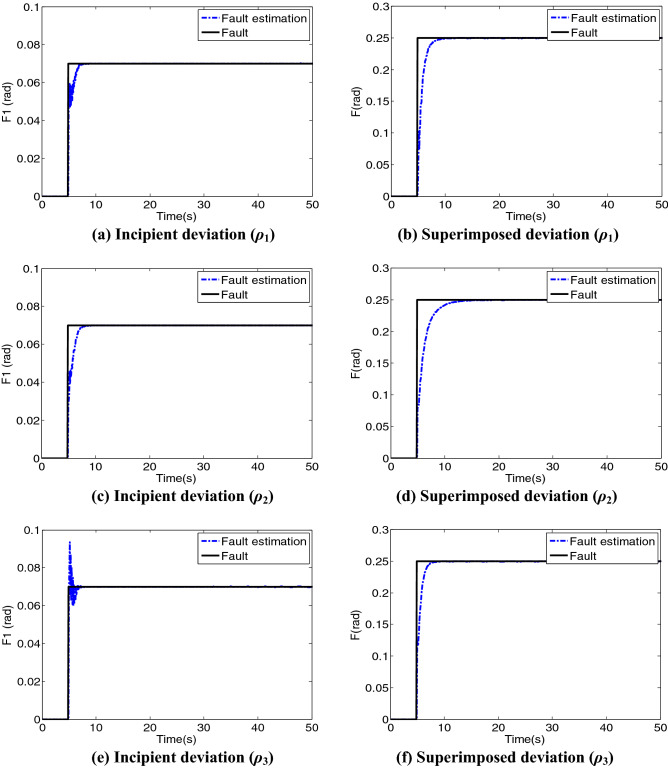
Fault estimation.

The estimation results of disturbance are substituted into system (22), whilst the effective results of fault estimation are shown in Fig. 7. The estimation curves of the three attitude angles (*ρ*_1_, *ρ*_2_, and *ρ*_3_) are slightly different. However, the incipient and total faults are well estimated.

**Figure 8:**
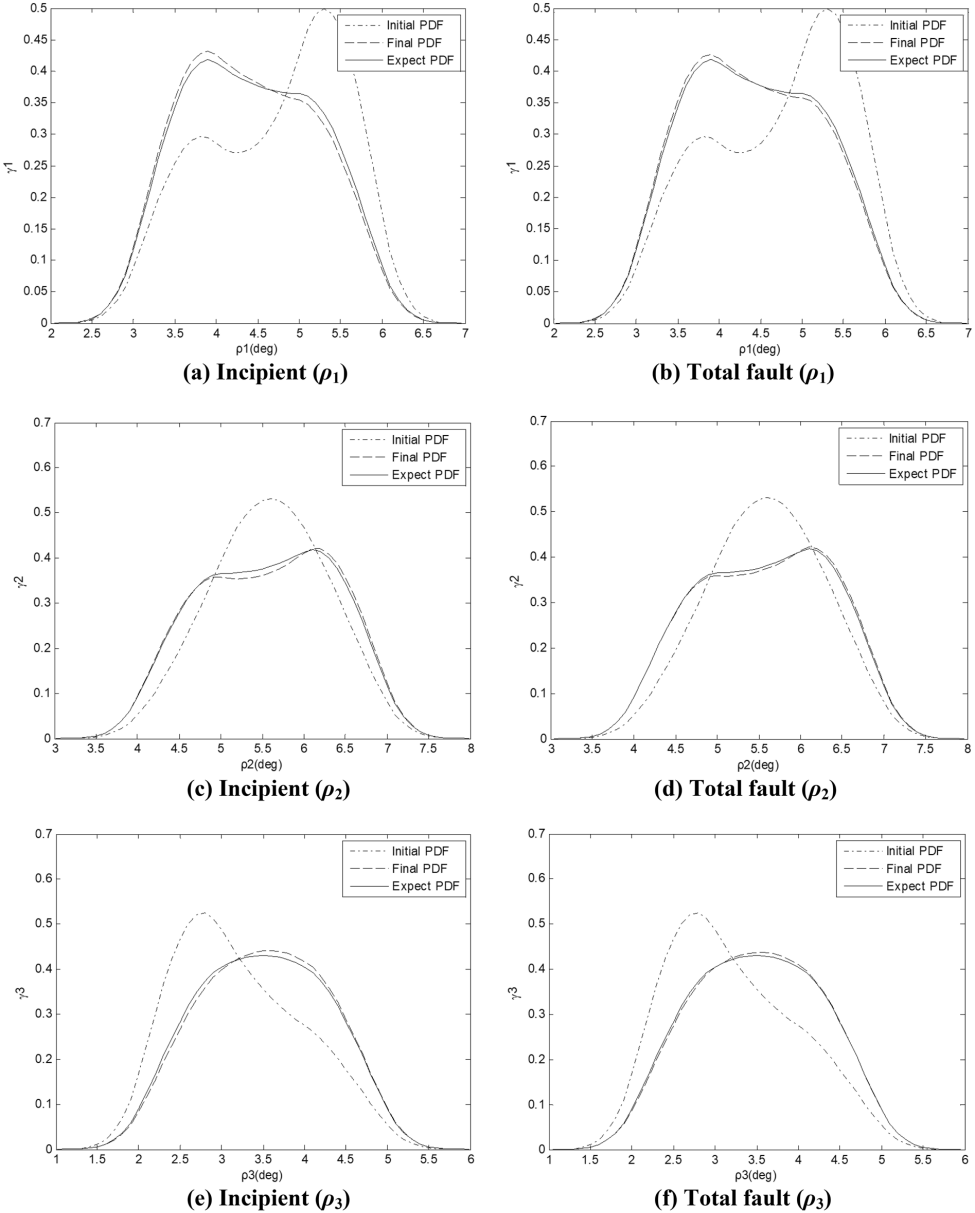
Expected and final PDFs.

Figure 8 shows the PDF plots of the stochastic attitude angle outputs under the prey adaptive tolerant controller (64). Fault-tolerant results show that prey adaptive controller can accurately track the ideal PDFs.

Experiment shows that for incipient faults and large deviation faults, the controller can limit the tracking errors and response times of the attitude PDFs in an acceptable ranges. Table 3 shows the PDF tracking errors in Fig. 8. The response time range is: 2–3.5 s. Based on Fig. 8, Table 4 statistics the performance and mean data of the PDFs. The mean values can be calculated by online simulation through formula (8).

**Table 4:** Fault tolerance tracking performance.

Performance	*ρ*_1_	*ρ*_2_	*ρ*_3_
Mean(*F*_1_/*F*)	4.383/4.388	5.774/5.768	3.682/3.685
t_res_(*F*_1_/*F*)	1.491/2.232	1.416/2.208	1.028/1.995
e_max_(*F*_1_/*F*)	0.014/0.019	0.011/0.017	0.011/0.014

In Table 4, t_res_ is the response time and e_max_ is the maximum steady-state error in the cross section. Angle unit: deg, and time unit: second. The tracking response time of an attitude mean is shorter than that of PDF, because the stabilization of the PDF shape needs more shape indicators to be stable at the same time, which takes longer, but can make the control better. The FTC algorithm has a good attitude tracking effect and can meet the engineering requirements of HFV.

## Conclusion

AN HFV model with attitude angle non-Gaussian uncertainty is proposed. This model uses PDFs to show more attitude angle information than the definitive mean and variance. HFV fault information and self-healing are determined through a change in the PDF shapes, thereby improving the fault self-healing and anti-laser capability. In double-fuzzy approximation linearized dynamics, the DOB effectively estimates the disturbance. Then a fault estimation observer is designed with the estimated disturbance to minimise the defined performance index. The strategy mimics the animal predation process successfully used to design controllers. Bionic prey adaptive FTC compensates for the effects of faults with different amplitudes on the three angle PDFs. The controller with no fault estimation has part of the direct repair function, thence estimation can help complete the repair without full fault amplitudes, the tolerance time is shorter than the estimation time. The output and ideal PDFs are consistent, good performance is verified in the simulation. This method constructs the control technology system of random aircraft, creates the condition for the design and implementation of anti-laser weapon, and has the frontier and application value.
